# SALL2 regulates neural differentiation of mouse embryonic stem cells through *Tuba1a*

**DOI:** 10.1038/s41419-024-07088-5

**Published:** 2024-09-30

**Authors:** Hui Xiong, Bowen Lin, Junyang Liu, Renhong Lu, Zheyi Lin, Chengwen Hang, Wenjun Liu, Lei Zhang, Jie Ding, Huixin Guo, Mingshuai Zhang, Siyu Wang, Zheng Gong, Duanyang Xie, Yi Liu, Dan Shi, Dandan Liang, Zhen Liu, Yi-Han Chen, Jian Yang

**Affiliations:** 1 State Key Laboratory of Cardiovascular Diseases, Shanghai East Hospital, School of Medicine, Tongji University, Shanghai, 200120 China; 2 Shanghai Arrhythmia Research Center, Shanghai East Hospital, School of Medicine, Tongji University, Shanghai, 200120 China; 3 Department of Cardiology, Shanghai East Hospital, School of Medicine, Tongji University, Shanghai, 200120 China; 4 Shanghai Frontiers Center of Nanocatalytic Medicine, Shanghai, 200092 China; 5 Department of Cell Biology, School of Medicine, Tongji University, Shanghai, 200092 China; 6 Clinical Center for Heart Research, Tongji University, Shanghai, 200092 China; 7 Department of Pathology and Pathophysiology, School of Medicine, Tongji University, Shanghai, 200092 China; 8 Institute of Neuroscience, CAS Center for Excellence in Brain Science and Intelligence Technology, CAS Key Laboratory of Primate Neurobiology, State Key Laboratory of Neuroscience, Chinese Academy of Sciences, Shanghai, 200031 China; 9 Shanghai Center for Brain Science and Brain-Inspired Intelligence Technology, Shanghai, China; 10 University of Chinese Academy of Sciences, Beijing, 100049 China; 11 Department of Anatomy, Histology and Embryology, School of Medicine, Tongji University, Shanghai, 200092 China; 12 Clinical Center for Brain and Spinal Cord Research, School of Medicine, Tongji University, Shanghai, 200092 China; 13 Department of Cardiology, the Second Hospital of Shanxi Medical University, Taiyuan, 030001 China; 14 Jinzhou Medical University, Jinzhou, Liaoning 121000 China; 15 Research Units of Origin and Regulation of Heart Rhythm, Chinese Academy of Medical Sciences, Shanghai, 200092 China

**Keywords:** Embryonic stem cells, Stem-cell differentiation, Differentiation

## Abstract

The *spalt* (*Sal*) gene family has four members (*Sall1-4*) in vertebrates, all of which play pivotal roles in various biological processes and diseases. However, the expression and function of SALL2 in development are still less clear. Here, we first charted SALL2 protein expression pattern during mouse embryo development by immunofluorescence, which revealed its dominant expression in the developing nervous system. With the establishment of *Sall2* deficient mouse embryonic stem cells (ESCs), the in vitro neural differentiation system was leveraged to interrogate the function of SALL2, which showed impaired neural differentiation of *Sall2* knockout (KO) ESCs. Furthermore, neural stem cells (NSCs) could not be derived from *Sall2* KO ESCs and the generation of neural tube organoids (NTOs) was greatly inhibited in the absence of SALL2. Meanwhile, transgenic expression of E1 isoform of SALL2 restored the defects of neural differentiation in *Sall2* KO ESCs. By chromatin immunoprecipitation sequencing (ChIP-seq), *Tuba1a* was identified as downstream target of SALL2, whose function in neural differentiation was confirmed by rescuing neural phenotypes of *Sall2* KO ESCs when overexpressed. In sum, by elucidating SALL2 expression dynamics during early mouse development and mechanistically characterizing its indispensable role in neural differentiation, this study offers insights into SALL2’s function in human nervous system development, associated pathologies stemming from its mutations and relevant therapeutic strategy.

## Introduction

The *spalt* (*Sal*) gene family consists of four members, *Sall1-4*, characterized by conserved zinc finger (ZF) domains. All the SAL proteins play an important role in the development of diseases [1]. SALL1 is essential for kidney development, evidenced by its homozygous deletion in mice leading to severe defects in the kidney, while in humans, SALL1 mutation causes Townes-Brocks syndrome, featuring anomalies in the ear, anus, kidney, and heart [2]. SALL3 deficient mouse manifests malformation in palate, tongue, and cranial nerves [3]. Also, SALL3 knockdown in human induced pluripotent stem cells (hiPSCs) inhibited endoderm and ectoderm differentiation [4]. SALL4 is one of the key pluripotency genes in maintaining pluripotency and self-renewal of ESCs, and its homozygous mutation in mouse is embryonic lethal during the peri-implantation stage [5]. SALL4 mutation in humans is related to Okihiro syndrome manifesting multiple organ defects and leukemia as well [6, 7].

*Sall2* is considered to be the most distant ortholog from the other three *Sal* members [8]. There are two promotors (P1, P2) in *Sall2* gene loci which generate *E1* and *E1A Sall2* isoforms, respectively, with 25 amino acids difference in their N-terminals [9, 10]. During mouse embryo development, in situ hybridization (ISH) assay showed that as early as embryonic day 7.5 (E7.5), *Sall2* mRNA was detected and consistently expressed until adulthood, concentrated in the developing brain, kidney, eyes, and ears, indicating that SALL2 may have an important association with the development of these organs, and SALL2 defect may cause corresponding congenital diseases [11, 12]. *Sall2* deficient mice may exhibit neural tube defect (NTD), depending on the genetic background, while compound deletion of *Sall1*, *Sall2*, and *Sall4* led to more severe NTD, implying functional redundancy among *Sal* family members [11]. It has also been reported that SALL2 mutation contributed to recessive ocular coloboma in human and mouse [13]. In addition, *Sall2* was identified as one of four transcription factors (TFs) reprogramming differentiated glioblastoma cells into stem-like tumor propagating cells (TPCs), suggesting its essential role in neural development and tumor propagation [14, 15]. However, the expression pattern of SALL2 during pre- and peri-implantation stage embryo development is still unclear, and the role of *Sall2* in early neural development has not been clarified.

Pluripotent stem cells (PSCs), either derived from early embryos or reprogrammed from somatic cells, have been widely used for mechanistic investigation of development and disease modeling [16, 17]. Moreover, by directed differentiation, numerous cell types can be generated from PSCs, thus providing excellent cell sources for regenerative medicine. With 2D culture, efficient differentiation protocols, such as for neurons, cardiomyocytes, and hepatic cells, have been developed [18, 19]. Recently, based on the self-organizing property of stem cells, using 3D culture to generate organoids mimicking in vivo organogenesis has been intensively investigated, which may facilitate both basic research and translational medicine [20, 21].

Here, we first aimed to complete the SALL2 protein expression pattern during early embryo development by immunofluorescence assay. Then we generated *Sall2* KO mouse ESC lines to investigate the role of *Sall2* in self-renewal and differentiation of ESCs. In particular, we systematically interrogated how *Sall2* affected neural differentiation, derivation of NSCs from ESCs and the formation of NTOs. Finally, by ChIP-seq, we identified *Tuba1a* as a downstream target of SALL2, involving in regulation of SALL2 mediated neural differentiation.

## Results

### Expression of SALL2 protein during mouse embryo development

We set to survey the expression of *Sall2* during mouse embryo development. First, we retrieved and analyzed the published RNA-seq datasets of mouse embryos from zygote to E15.5 (GSE222357, GSE98150, GSE214161, GSE216492, and https://www.informatics.jax.org/gxd/marker/MGI:1354373), which showed that the transcription of *Sall2* initiated at E5.5 and was restricted to the epiblast of the embryo (Supplementary Fig. S1A). With embryo development, the expression of *Sall2* gradually increased and was mainly detected in the brain, kidney, and other tissues (Supplementary Fig. S1B). These results were consistent with the reported ISH of *Sall2* mRNA expression during mouse embryo development [11]. Next, in order to determine SALL2 protein expression during mouse embryo development, we applied immunofluorescence staining of SALL2 from zygote to E15.5 embryos.

**Fig. 1:**
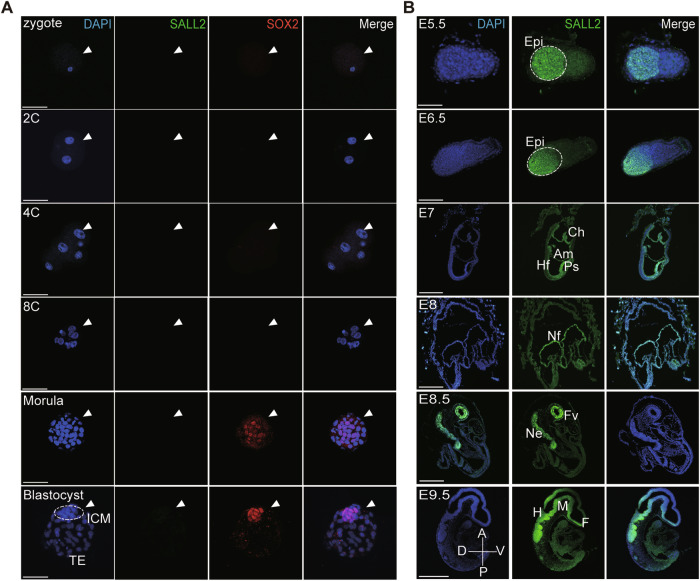
The expression pattern of SALL2 protein in mouse E0.5-E15.5 embryos. **A** Expression of SALL2 from mouse zygote to blastocyst. The embryos were immunostained for SALL2 and SOX2. DAPI stained the nuclei. The arrowheads indicated the embryo, and the dashed circle outlined the ICM of the blastocyst. Scale bar, 50 μm. **B** Expression of SALL2 in mouse E5.5-E9.5 embryos. The embryos were immunostained for SALL2. DAPI stained the nuclei. Dashed circles outlined the Epi of embryos. E5.5, scale bar, 75 μm; E6.5, scale bar, 250 μm; E7-E9.5, scale bar, 250 μm. C cell, ICM inner cell mass, TE trophectoderm, Epi epiblast, Ch chorion, Am amnion, Hf headfold, Ps primary streak, Nf neural fold, Fv forebrain vesicle, Ne neural ectoderm, F forebrain, M midbrain, H hindbrain, A anterior, P posterior, D dorsal, V ventral.

For preimplantation embryo, from zygote to blastocyst, there was no SALL2 protein expression observed (Fig. 1A). After implantation, SALL2 was detected in the epiblast of E5.5-E6.5 embryos, then gradually increased (Fig. 1B). At gastrulation stage embryo (E7), SALL2 mainly expressed in neuroectoderm and primitive streak, and a few SALL2^+^ cells were detected in amnion and chorion (Fig. 1B). In E8-E8.5 embryos, a critical period for the formation and closure of neural tube (NT) [22], SALL2 was extensively expressed in neural fold, neuroectoderm, forebrain vesicle and NT (Fig. 1B and Supplementary Fig. S1C, F). From E9.5-E10.5, constant SALL2 protein was detected in the nervous system, such as NT and brain (Fig. 1B and Supplementary Fig. S1C, F).

It has been reported that in the cerebral cortex of the adult mouse, SALL2 was translocated from the nucleus into the cytoplasm [23]. Interestingly, for E11.5 embryos, in the midbrain limbic system, the cytoplasm expression of SALL2 was observed in neural cells characterized by the expression of neural marker TUBB3. Again, SALL2 is mainly expressed in the forebrain, diencephalon, midbrain, hindbrain, and NT (Supplementary Fig. S1D, E, G). At E12.5, except its expression in the telencephalon, midbrain, and pons, SALL2 is also expressed in the spinal cord and preoptic neuroepithelium (Supplementary Fig. S1D, G).

With the development and maturation of organs in mouse embryo, from E13.5 to E15.5, SALL2 expression decreased with its expression pattern being stable in telencephalon, diencephalon, midbrain, pons, hindbrain, spinal cord, preoptic neuroepithelium as well as pituitary gland and olfactory epithelium (Supplementary Fig. S1D, E, G).

Therefore, by combining immunofluorescence staining results with RNA-seq data, we charted a complete SALL2 expression pattern during mouse embryo development (E0.5-E15.5) and found that SALL2 mainly expressed in the developing nervous system, indicating its potential role in neurogenesis.

### Impaired pluripotency of *Sall2* deficient ESCs

PSCs have been widely used as in vitro model [24] to investigate gene function in development and diseases. To interrogate the role of SALL2 in early embryo development, we first examined SALL2 expression in ESCs, while there was almost no SALL2 expression in naïve ESCs cultured in 2i/LIF, ESCs cultured in serum/LIF medium (M15), which contain differentiating cells, expressed SALL2 (Supplementary Figs. S1H, S2C). These results mirrored our in vivo SALL2 immunostaining data, as naïve ESCs resemble epiblast in blastocyst without SALL2 expression, while SALL2 was detected in post-implantation embryos [25, 26] (Fig. 1A, B).

**Fig. 2:**
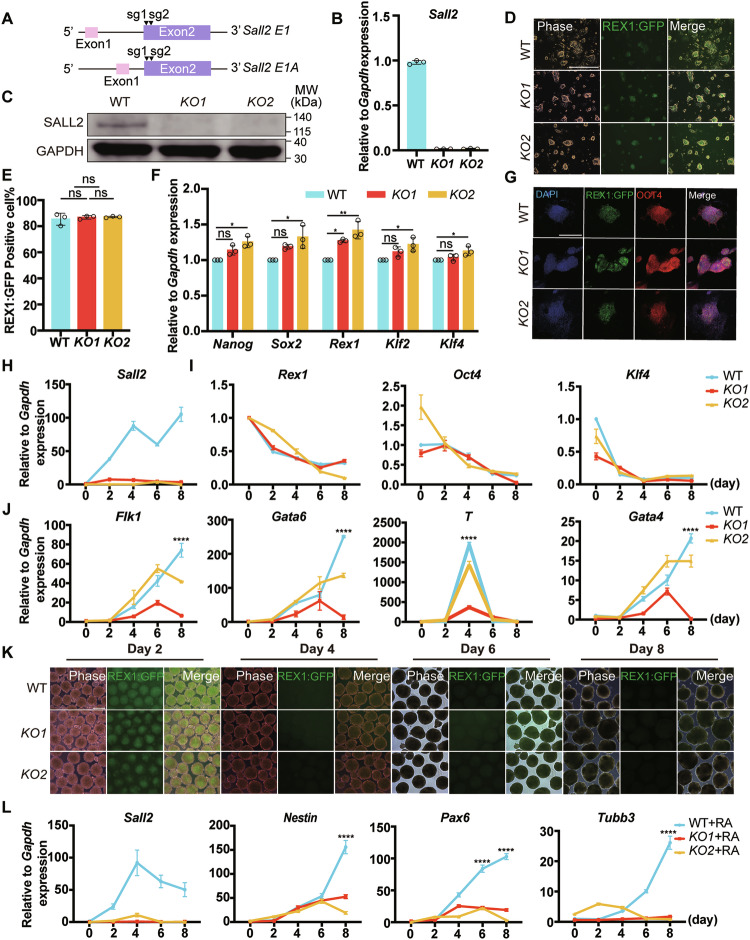
Construction and characterization of REX1:GFP *Sall2* KO ESC lines. **A** Schematic diagram of sgRNA design for *Sall2* deletion. **B** mRNA expression of *Sall2* in REX1:GFP *Sall2* KO and WT ESCs. Relative to *Gapdh* expression (*n* = 3, technical replicates). **C** SALL2 protein expression in REX1:GFP *Sall2* KO and WT ESCs. GAPDH served as a loading control. **D** Morphological analysis of REX1:GFP *Sall2* KO ESCs. Scale bar, 250 μm. **E** Flow cytometry analysis of REX1:GFP^+^ cells in REX1:GFP *Sall2* KO ESCs (*n* = 3, biological replicates). Statistical significance was determined by one-way ANOVA with Tukey’s test. ns not significant. **F** qRT-PCR analysis of pluripotency markers (*Nanog*, *Sox2*, *Rex1*, *Klf2*, *Klf4*) in REX1:GFP *Sall2* KO and WT ESCs. Relative to *Gapdh* expression (*n* = 3, technical replicates). Statistical significance was determined by two-way ANOVA with Tukey’s test. *P < 0.05, **P < 0.01, ns not significant. **G** Expression of OCT4 in REX1:GFP *Sall2* KO and WT ESCs. The cells were immunostained for OCT4. DAPI stained nuclei. Scale bar, 250 μm. **H** qRT-PCR analysis of *Sall2* in REX1:GFP *Sall2* KO and WT ESCs during EB differentiation. Relative to *Gapdh* expression (*n* = 3, technical replicates). **I** qRT-PCR analysis of pluripotency markers (*Rex1*, *Oct4*, *Klf4*) during EB differentiation. Relative to *Gapdh* expression (*n* = 3, technical replicates). **J** qRT-PCR analysis of meso-endoderm markers (*Flk1*, *Gata6*, *T*, *Gata4*) during EB differentiation. Relative to *Gapdh* expression (*n* = 3, technical replicates). Statistical significance was determined by two-way ANOVA with Tukey’s test, indicating significant changes between *Sall2* KO cell lines (*KO1*, *KO2*) and WT cells. ****P < 0.0001. **K** Observation of REX1:GFP during RA-induced EB differentiation in REX1:GFP *Sall2* KO and WT ESCs. Scale bar, 250 μm. **L** qRT-PCR analysis of ectoderm markers during RA-induced EB differentiation. Relative to *Gapdh* expression (*n* = 3, technical replicates). Statistical significance was determined by two-way ANOVA with Tukey’s test, indicating significant changes between *Sall2* KO cell lines (*KO1* + RA, *KO2* + RA) and WT cells (WT + RA). ****P < 0.0001.

The pluripotency marker REX1 tagged GFP (REX1:GFP) ESC reporter line has been broadly used to monitor the self-renewal and pluripotency of ESCs [27, 28]. We applied clustered regularly interspaced short palindromic repeats (CRISPR)/Cas9 technology to knock out *Sall2* in REX1:GFP ESCs [29]. Since at least two *Sall2* isoforms have been discovered, we designed guide RNA (gRNA) targeting the common Exon 2 of *Sall2* (Fig. 2A). After transfection and drug selection, ESC colonies were picked up for characterization. By Sanger sequencing, we verified at least two independent *Sall2* KO ESC lines with different deletions (Supplementary Fig. S2A, B). Results of qRT-PCR, western blot, and immunofluorescence staining showed *Sall2* deletion in these lines (Fig. 2B, C and Supplementary Fig. S2C). Moreover, by checking the predicted off-target sites, no mutation was detected (Supplementary Fig. S2D). Thus, we have successfully established *Sall2* KO REX1:GFP ESC lines (REX1:GFP *Sall2* KO).

Next, we characterized the REX1:GFP *Sall2* KO ESC lines. Morphologically, the *Sall2* KO ESCs displayed a dome shape, similar to wild-type (WT) ESCs (Fig. 2D). When observed under fluorescence microscope, the REX1:GFP expression in *Sall2* KO ESCs was comparable to WT cells, which was further confirmed by flow cytometry analysis of REX1:GFP (Fig. 2D, E). We then determined the expression of some pluripotency markers *Nanog, Sox2, Rex1, Klf2*, and *Klf4*. For *Nanog*, *Sox2*, *Klf2*, and *Klf4* expression, there was no significant difference between WT and *KO1* ESCs, while a slight increase was detected in *KO2* ESCs; *Rex1* expression increased moderately in both *KO1* and *KO2* ESCs (Fig. 2F). The difference in pluripotency gene expression may be attributed to clone difference [30, 31]. Meanwhile, by immunostaining, the OCT4 protein expression was not affected in *Sall2* KO ESCs as well (Fig. 2G).

We then tested whether *Sall2* KO ESC lines could be established from blastocysts with *Sall2* deficiency. Zygotes were injected with *Sall2* single guide RNA (sgRNA) and Cas9 protein, then cultured in vitro until the blastocyst stage [32, 33]. From 91 zygotes injected, 77 progressed to blastocysts (84.6%), as confirmed by SOX2 expression in the inner cell mass (ICM), which was not significantly lower than that of the noninjected embryos (92.3%) (Supplementary Fig. S2E–G). Meanwhile, eight embryos were collected for PCR and sequencing, which showed about 94.2% KO efficiency (Supplementary Fig. S2H, I). From 16 blastocysts cultured in M15, nine outgrowths formed, after passaging, nine ESC clones were established (56.25%), all of which showed *Sall2* deletion (Supplementary Fig. S2J–L). We further performed qRT-PCR and immunostaining to check the expression of pluripotency markers, which displayed comparable levels between WT and *Sall2* KO ESCs, though no SALL2 protein was detected (Supplementary Fig. S2M, N). These data demonstrated that SALL2 was not required for the acquisition of ESC state.

As *Sall2* expression increased in heterogenous ESC culture with M15 and in post-implantation embryos, we performed embryoid body (EB) differentiation to determine the effects of *Sall2* on the pluripotency of ESCs [34, 35]. Hang-drop protocol was applied to form EBs [36, 37], during the differentiation, *Sall2* expression increased in WT cells, and the expression of *Rex1*, *Oct4*, and *Klf4* reduced in both *Sall2* KO and WT cells (Fig. 2H, I). However, the meso-endoderm markers *Flk1*, *Gata6, T*, and *Gata4* significantly decreased in *Sall2* KO cells (Fig. 2J). As the differentiated cells in hang-drop EBs were mainly from meso-endoderm lineage, we treated the EBs with retinoic acid (RA), which efficiently induces neuroectoderm differentiation [38, 39]. Upon RA induction, the REX1:GFP fluorescence gradually faded away in both WT and *Sall2* KO cells, meanwhile, the neural markers *Nestin*, *Pax6* and *Tubb3* significantly upregulated in WT cells, in contrast, in *Sall2* KO cells, their expression was still at very low level (Fig. 2K, L). Together, these data indicated that *Sall2* deficiency impaired the pluripotency of ESCs.

### *Sall2* was critical for the neural differentiation of ESCs

**Fig. 3:**
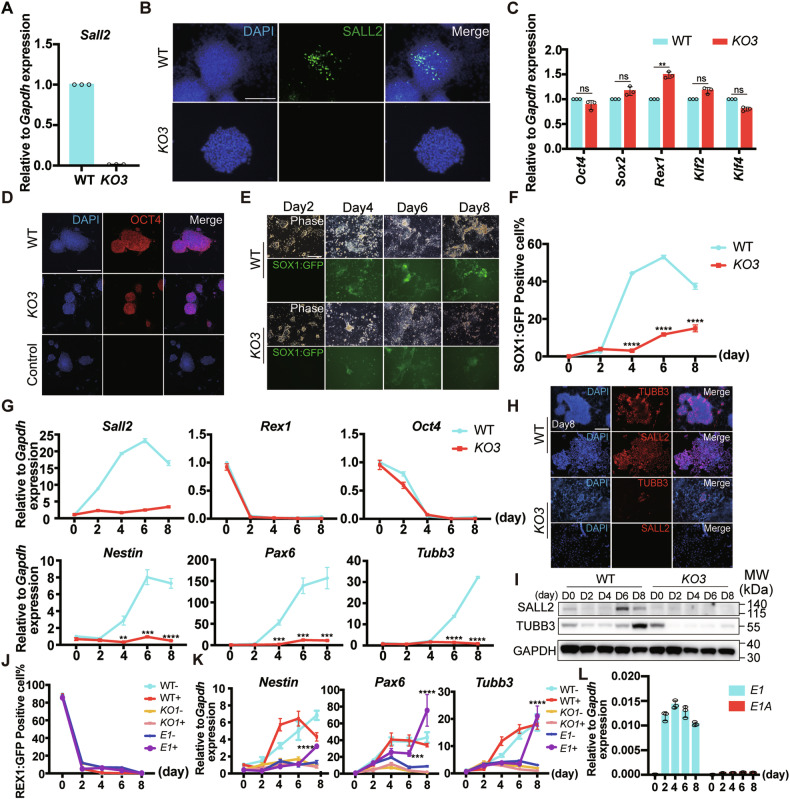
*Sall2* KO inhibited neural differentiation of ESCs. **A** mRNA expression of *Sall2* in SOX1:GFP *Sall2* KO ESCs. Relative to *Gapdh* expression (*n* = 3, technical replicates). **B** Expression of SALL2 in SOX1:GFP *Sall2* KO and WT ESCs. The cells were immunostained for SALL2. DAPI stained nuclei. Scale bar, 250 μm. **C** qRT-PCR analysis of pluripotency markers (*Oct4*, *Sox2*, *Rex1*, *Klf2*, *Klf4*) in SOX1:GFP *Sall2* KO and WT ESCs. Relative to *Gapdh* expression (*n* = 3, technical replicates). Statistical significance was determined by an unpaired *t*-test. **P < 0.01, ns not significant. **D** Expression of OCT4 in SOX1:GFP *Sall2* KO and WT ESCs. The cells were immunostained for OCT4. DAPI stained nuclei. Scale bar, 250 μm. Cells treated with secondary antibodies only served as control. **E** Phase and fluorescence images of SOX1:GFP *Sall2* KO and WT ESCs during monolayer neural differentiation. Scale bar, 250 μm. **F** Flow cytometry analysis of SOX1:GFP^+^ cells during monolayer neural differentiation of *Sall2* KO and WT ESCs (*n* = 3, biological replicates). Statistical significance was determined by an unpaired *t*-test. ****P < 0.0001. **G** qRT-PCR analysis of *Sall2*, pluripotency markers (*Rex1*, *Oct4*), and neuroectoderm markers (*Nestin*, *Pax6*, *Tubb3*) during monolayer neural differentiation of SOX1:GFP *Sall2* KO and WT ESCs. Relative to *Gapdh* expression (*n* = 3, technical replicates). Statistical significance was determined by unpaired *t*-test. **P < 0.01, ***P < 0.001, ****P < 0.0001. **H** Expression of TUBB3 and SALL2 during monolayer neural differentiation of SOX1:GFP *Sall2* KO and WT ESCs at day 8. The cells were immunostained for TUBB3 and SALL2. DAPI stained nuclei. Scale bar, 250 μm. **I** Western blot analysis of SALL2 and TUBB3 expression during monolayer neural differentiation of SOX1:GFP *Sall2* KO and WT ESCs at days 2, 4, 6, and 8. GAPDH served as a loading control. **J** Flow cytometry analysis of REX1:GFP^+^ cells in REX1:GFP OE-*E1*, *KO1*, and WT ESCs with or without DOX induction during monolayer neural differentiation (*n* = 3, biological replicates). **K** qRT-PCR analysis of neural markers (*Nestin*, *Pax6*, *Tubb3*) in REX1:GFP OE-*E1*, *KO1*, and WT ESCs with or without DOX induction during monolayer neural differentiation. Relative to *Gapdh* expression (*n* = 3, technical replicates). Statistical significance was determined by two-way ANOVA with Tukey’s test, indicating significant changes between REX1:GFP OE-*E1* cells with (*E1*+) and without (*E1*−) DOX induction. ***P < 0.001, ****P < 0.0001. **L** mRNA Expression of *E1*, *E1A Sall2* in REX1:GFP WT ESCs during monolayer neural differentiation. Relative to *Gapdh* expression (*n* = 3, technical replicates).

Regarding SALL2, mainly expressed in the nervous system during embryo development and *Sall2* KO EBs manifested impaired neural differentiation, we next focus on its role in neural differentiation [40]. To this end, we selected the SOX1:GFP ESC line to perform monolayer neural differentiation. *Sox1* is a key neural progenitor marker, by monitoring SOX1:GFP expression, the neural differentiation efficiency can be determined conveniently [41, 42]. First, we generated SOX1:GFP *Sall2* KO ESC lines by utilizing the same strategy as we did for REX1:GFP *Sall2* KO ESCs. The deletion of the *Sall2* coding sequence was confirmed by sequencing the *Sall2* loci (Supplementary Fig. S3A, B). Then, the mRNA and protein level of *Sall2/*SALL2 was determined by qRT-PCR, immunostaining, and western blot, which showed deficient *Sall2* expression (Fig. 3A, B and Supplementary Fig. S3C). Similarly, pluripotency markers were not significantly affected in SOX1:GFP *Sall2* KO ESCs (Fig. 3C, D).

We then performed neural differentiation with SOX1:GFP *Sall2* KO ESCs. By observing the GFP signal under a fluorescence microscope, we found the SOX1:GFP^+^ cells were significantly fewer in *Sall2* KO cells than in WT cells (Fig. 3E). We further quantified the dynamic change of SOX1:GFP^+^ population during differentiation by flow cytometry. As early as day 4, some SOX1:GFP^+^ cells emerged in WT cells, whereas no GFP signal was observed in *Sall2* KO cells. Next, the SOX1:GFP^+^ population reached the peak on day 6, and was relatively stable from days 6 to 8 in WT cells, while SOX1:GFP still weakly expressed during the neural differentiation in *Sall2* KO cells (Fig. 3F).

Meanwhile, we examined the expression of *Sall2*, pluripotency and lineage marker genes during differentiation. In WT cells, *Sall2* mRNA consistently increased until day 6, then went down at day 8, similarly, high SALL2 protein level was detected at day 4 (Fig. 3G, I). The expression of *Rex1* and *Oct4* quickly lost in both *Sall2* KO and WT cells. However, the expression level of *Nestin*, *Pax6*, and *Tubb3* was not upregulated in *Sall2* KO cells, consistent with RA-induced EBs differentiation (Fig. 3G). Also, both immunostaining and western blot confirmed the reduction of TUBB3 expression in *Sall2* KO cells at differentiation day 8 (Fig. 3H, I).

To corroborate these findings, we further performed monolayer neural differentiation with REX1:GFP *Sall2* KO ESC lines. The REX1:GFP^+^ population diminished quickly during the first 2 days of differentiation, as revealed by flow cytometry (Supplementary Fig. S3D). Concomitant with SOX1:GFP ESCs, the *Sall2* level increased during the differentiation of REX1:GFP ESCs (Supplementary Fig. S3E). While the transcription level of *Rex1* and *Oct4* reduced in both *Sall2* KO and WT cells, again, low expression of *Nestin*, *Pax6*, and *Tubb3* was detected in *Sall2* KO cells (Supplementary Fig. S3F, G). Also, by immunostaining, very fewer TUBB3^+^ cells were observed in *Sall2* KO cells at differentiation day 8 (Supplementary Fig. S3H). Thus, the impaired neural differentiation due to *Sall2* deletion was validated in two independent *Sall2* KO ESC lines.

### *E1* isoform of *Sall2* restored neural differentiation of *Sall2* KO ESCs

To further validate the function of *Sall2* in neural differentiation, we designed rescue experiments by transgenic overexpression of *Sall2* in REX1:GFP *Sall2* KO ESCs. We constructed PB-TRE-*E1*, PB-TRE-*E1A* vectors, the expression of these two *Sall2* isoforms can be induced by doxycycline (DOX) [10, 43, 44] (Supplementary Fig. S3I). After transfection and colony picking, the integration of transgene was confirmed by genomic PCR and western blot verified transgenic *Sall2* expression in the presence of DOX (Supplementary Fig. S3J, K). We named the transgenic *Sall2* expressing cells as REX1:GFP OE-*E1* and REX1:GFP OE-*E1A* ESCs, respectively.

We then set up neural differentiation for REX1:GFP WT, *Sall2* KO, OE-*E1*, and OE-*E1A* ESCs with and without DOX. Flow cytometry analysis showed that during neural differentiation, REX1:GFP^+^ populations decreased significantly in 2 days in all groups (Fig. 3J and Supplementary Fig. S3L). In terms of neural markers, intriguingly, upon *E1 Sall2* expression, the level of *Nestin*, *Pax6*, and *Tubb3* was comparable to that of WT cells at differentiation day 8 (Fig. 3K), whereas, overexpressing *E1A Sall2* did not elevate their expression (Supplementary Fig. S3M). We therefore analyzed the expression of *E1* and *E1A* isoforms during neural differentiation, which showed that *E1* level was much higher than *E1A* (Fig. 3L). That may be one of the reasons that only *E1* overexpression can restore defected neural differentiation of *Sall2* KO ESCs. Taken together, *Sall2* played a critical role during neural differentiation from ESCs and *E1* isoform may be the main determinant.

### SALL2 was indispensable for the derivation of NSCs from ESCs

SOX1 expression was low in *Sall2* KO cells during neural differentiation, and SALL2 was one of the factors that can reprogram differentiated glioma cells into glioma stem cells [14, 15]. During early neural development, NSCs played an important role in neural lineage differentiation [45, 46]. NSCs have the ability to proliferate, migrate, and differentiate into neurons, astrocytes, and oligodendrocytes, providing cell sources for nerve repair [47, 48]. We speculated that *Sall2* may participate in the establishment of NSCs. We thus leveraged the protocol for NSCs derivation from ESCs on SOX1:GFP *Sall2* KO ESCs.

**Fig. 4:**
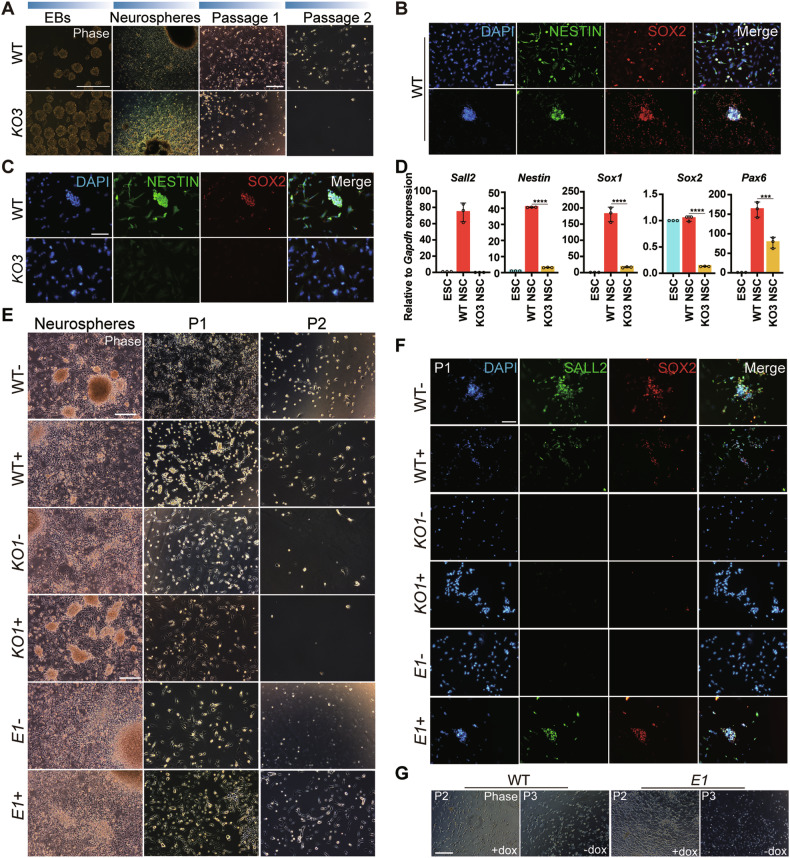
*Sall2* was indispensable for the derivations of NSCs from ESCs. **A** Phase images of NSCs derivation from SOX1:GFP *Sall2* KO and WT ESCs. EBs and Neurospheres, scale bar, 500 μm; P1 and P2, scale bar, 250 μm. **B** Expression of NESTIN and SOX2 in NSCs (P7) derived from SOX1:GFP WT ESCs. DAPI stained nuclei. Scale bar, 250 μm. **C** Expression of NESTIN and SOX2 in NSCs (P1) derived from SOX1:GFP *Sall2* KO and WT ESCs. The cells were immunostained for NESTIN and SOX2. DAPI stained nuclei. Scale bar, 250 μm. **D** qRT-PCR analysis of *Sall2* and NSC markers (*Nestin*, *Sox1*, *Sox2*, *Pax6*) in NSCs (P1) derived from SOX1:GFP *Sall2* KO and WT ESCs. Relative to *Gapdh* expression (*n* = 3, technical replicates). Statistical significance was determined by one-way ANOVA with Tukey’s test. ***P < 0.001, ****P < 0.0001. **E** Phase images of NSCs derivation from REX1:GFP OE-*E1*, *KO1,* and WT ESCs with or without DOX induction. Scale bar, 250 μm. **F** Expression of SALL2 and SOX2 in NSCs (P1) derived from REX1:GFP OE-*E1*, *KO1*, and WT ESCs with or without DOX induction. The cells were immunostained for SALL2 and SOX2. DAPI stained nuclei. Scale bar, 250 μm. **G** Phase images of NSCs derived from REX1:GFP OE-*E1* and WT before and after DOX removal (P2, P3). Scale bar, 250 μm.

After 3 days of suspension culture, neural spheres formed, which were then transferred into an adhesive plate. When NSCs migrated out from the neurospheres, the cells were dissociated and plated into an NSC medium for expansion and characterization [49]. NSCs derived from SOX1:GFP WT ESCs displayed elongated morphology whereas the cells from SOX1:GFP *Sall2* KO ESCs were more differentiated with large and flat morphology (Fig. 4A). Subsequently, after second passage, the *Sall2* KO cells all died while WT NSCs continuously grew and could be passaged; also, the expression of NSC markers, NESTIN and SOX2, confirmed their identity (Fig. 4A, B). To understand this phenotype, we performed immunostaining of NESTIN and SOX2 on cells after the first passage. Surprisingly, no NESTIN and SOX2 expression was observed in *Sall2* KO cells, indicating that in the absence of *Sall2*, NSCs could not be derived from ESCs (Fig. 4C). We then performed qRT-PCR to characterize the passage 1 (P1) of WT and *Sall2* KO cells. After *Sall2* KO, the expression of *Nestin*, *Sox1*, *Sox2*, and *Pax6* were repressed (Fig. 4D). We also tried to derive NSCs from REX1:GFP *Sall2* KO ESCs. Similarly, at P1, the *Sall2* KO cells lost NESTIN and SOX2 expression, then died after the second passage, while NSCs were successfully derived from REX1:GFP WT ESCs, verified by SOX2 expression and the expression of *Nestin*, *Sox1*, *Sox2* and *Pax6* (Supplementary Fig. S4A–C).

In order to further verify the function of SALL2 in NSCs, we conducted rescue experiments by overexpressing *E1 Sall2* in REX1:GFP *Sall2* KO ESCs. During NSCs derivation, at P1, there were NESTIN and SOX2 positive cells upon DOX induction, which could be passaged steadily (Fig. 4E, F). However, when *E1 Sall2* transgene expression was shut off after second passage, the cells gradually died (Fig. 4G). In all, *E1 Sall2* played a vital role in the derivation of NSCs from ESCs.

### SALL2 deletion impacted NTOs generation

Defects in NT development have been documented in *Sall2* KO mice [11], we also showed that *Sall2* was essential for NSCs derivation from ESCs. To get insights of *Sall2* in NT development, we generated NTOs from ESCs as previously reported [50].

**Fig. 5:**
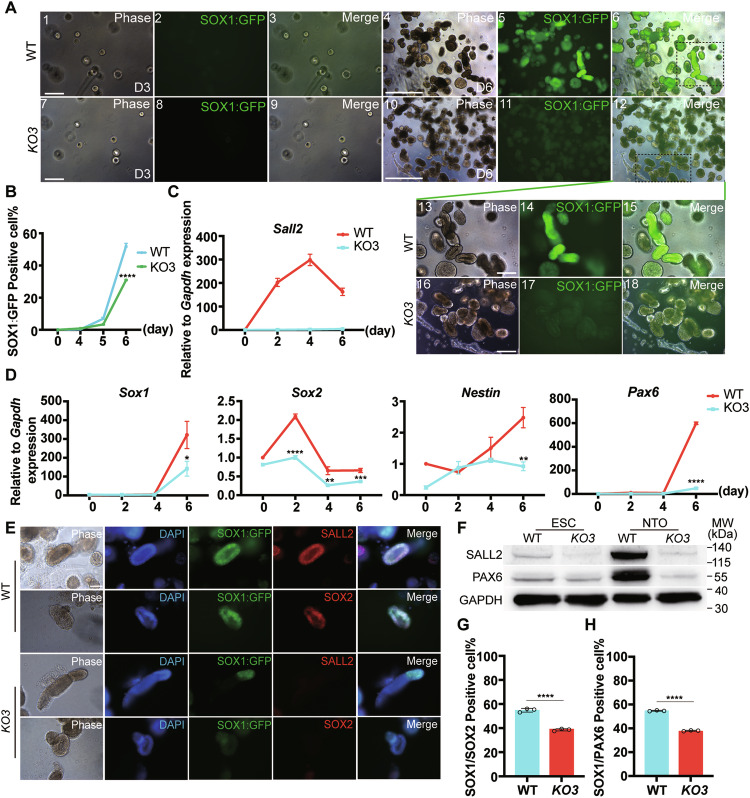
*Sall2* impaired NTOs formation from ESCs. **A** Phase and fluorescence images of NTOs formation from SOX1:GFP *Sall2* KO and WT ESCs. The dashed box indicated enlarged images. Scale bar, 500 μm (4–6, 10–12); 250 μm (1–3, 7–9, 13–18). **B** Flow cytometry analysis of SOX1:GFP^+^ cells during NTOs formation from SOX1:GFP *Sall2* KO and WT ESCs at days 4, 5, 6 (*n* = 3, biological replicates). Statistical significance was determined by unpaired *t*-test. ****P < 0.0001. **C** *Sall2* expression during NTOs formation from SOX1:GFP *Sall2* KO and WT ESCs at days 2, 4, 6. Relative to *Gapdh* expression (*n* = 3, technical replicates). **D** qRT-PCR analysis of NTO markers (*Sox1*, *Sox2*, *Nestin*, *Pax6*) during NTOs formation from SOX1:GFP *Sall2* KO and WT ESCs at days 2, 4, 6. Relative to *Gapdh* expression (*n* = 3, technical replicates). Statistical significance was determined by unpaired *t-*test. *P < 0.05, **P < 0.01, ***P < 0.001, ****P < 0.0001. **E** Immunofluorescence of SALL2 and SOX2 expression in SOX1:GFP *Sall2* KO and WT NTOs at differentiation day 6. The NTOs were immunostained for SALL2 and SOX2. DAPI stained nuclei. Scale bar, 250 μm. **F** Western blot analysis of PAX6 and SALL2 expression in SOX1:GFP *Sall2 KO3* and WT NTOs at differentiation day 6. GAPDH served as a loading control. **G** Flow cytometry analysis of SOX1:GFP/SOX2 positive cells in SOX1:GFP *Sall2* KO and WT NTOs at differentiation day 6 (*n* = 3, biological replicates). Statistical significance was determined by unpaired *t*-test. ****P < 0.0001. **H** Flow cytometry analysis of SOX1:GFP/PAX6 positive cells in SOX1:GFP *Sall2* KO and WT NTOs at differentiation day 6 (*n* = 3, biological replicates). Statistical significance was determined by unpaired *t*-test. ****P < 0.0001.

The naïve ESCs were transiently converted to primed epiblast stem cells (EpiSCs), then differentiated to NSCs and self-organized into NTOs. At day 5, the NT like morphology was observed in both WT and SOX1:GFP *Sall2* KO ESCs accompanied by some SOX1:GFP^+^ cells. At day 6, the WT cells formed a long, narrow tube-like structure resembling NT, with SOX1:GFP^+^ cells clustering in NTOs (Fig. 5A). However, the number of NTOs as well as SOX1:GFP^+^ cells were fewer in *Sall2* KO cells compared with WT cells under the microscope, which was further validated by tracking SOX1:GFP expression with flow cytometry. When measuring SOX1:GFP and *Sall2* transcription at different time points (days 4, 5, and 6) during NTOs formation, *Sall2* gradually increased and reached a peak at day 4 and then decreased (Fig. 5B, C). Moreover, the level of *Sox1*, *Sox2*, *Nestin*, and *Pax6* was considerably lower in *Sall2* KO NTOs (Fig. 5D). Additionally, immunofluorescence assay showed the co-localization of SALL2/SOX1:GFP and SOX1:GFP/SOX2 in WT NTOs while fewer positive signals were detected in *Sall2* KO NTOs (Fig. 5E). Meanwhile, we collected WT and *Sall2* KO NTOs and determined PAX6 protein expression by western blot, which also showed that PAX6 was much lower in *Sall2* KO NTOs (Fig. 5F).

We then dissociated WT and *Sall2* KO NTOs into single cells to perform flow cytometry analysis. The percentages of SOX1:GFP/SOX2 and SOX1:GFP/PAX6 positive cells in WT NTOs were significantly higher than those of *Sall2* KO NTOs (Fig. 5G, H). Consistently, fewer SOX1:GFP and SOX1:GFP/SALL2 positive cells were detected in *Sall2* KO NTOs (Supplementary Fig. S5A).

The capability of generating NTOs was also examined on REX1:GFP *Sall2* KO ESCs. In line with SOX1:GFP ESCs, fewer NTOs formed from REX1:GFP *Sall2* KO ESCs, and the expression of *Sox1*, *Sox2*, and *Pax6* decreased as well (Supplementary Fig. S5B). By immunostaining, the expression of SOX2 was much lower in *Sall2* KO NTOs as well (Supplementary Fig. S5C). We then induced *Sall2* transgene expression during NTOs generation. By qRT-PCR, *E1 Sall2* led to increased levels of *Sox1* and *Pax6* (Supplementary Fig. S5D). Meanwhile, we performed flow cytometry analysis of REX1:GFP *Sall2 E1* overexpressing NTOs, which showed increased percentages of SOX2, PAX6, and SOX2/PAX6 positive cells (Supplementary Fig. S5E). These data not only verified the previously reported NTDs in *Sall2* KO mice, but also demonstrated the indispensable role of *Sall2* in NT development.

### *Tuba1a* mediated SALL2 regulated neural differentiation

**Fig. 6:**
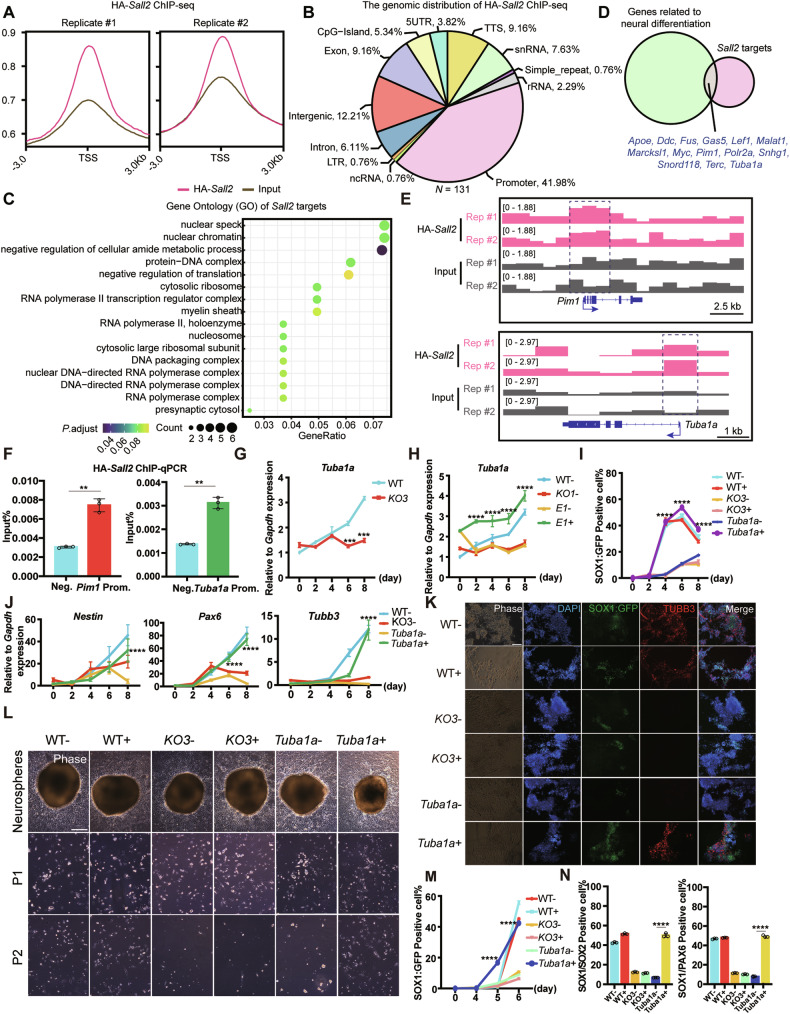
SALL2 regulated neural differentiation of ESCs through *Tuba1a.* **A** Metagene showing ChIP-seq signal profiles of HA-*Sall2* (pink) and Input (gray) around the TSS regions (±3.0 Kb). Each group has two replicates. **B** The genomic distribution of HA-*Sall2* binding regions identified by HA-*Sall2* ChIP-seq. **C** GO analysis of SALL2 targets identified by HA-*Sall2* ChIP-seq. **D** Venn diagram of overlaps of SALL2 binding genes (*Sall2* targets) with genes related to neural differentiation. **E** Genome browser view of HA-*Sall2* ChIP-seq analysis in HA-*Sall2* (pink) and Input (gray) samples at *Pim1* and *Tuba1a* gene loci. The promoter regions of *Pim1* and *Tuba1a* were marked by dashed boxes. **F** ChIP-qPCR analysis of *Pim1* and *Tuba1a*. The binding of SALL2 to the promoter region of *Pim1* (*Pim1* prom.) and *Tuba1a* (*Tuba1a* prom.) was measured by ChIP-qPCR, and the intergenic nonbinding region was amplified as negative control (Neg.). Relative to 5% input (*n* = 3, technical replicates). Statistical significance was determined by unpaired *t*-test. **P < 0.01. **G** Expression of *Tuba1a* during monolayer neural differentiation of SOX1:GFP Sall2 KO and WT ESCs. Relative to *Gapdh* expression (*n* = 3, technical replicates). Statistical significance was determined by unpaired *t*-test. ***P < 0.0001. **H** Expression of *Tuba1a* during monolayer neural differentiation of REX1:GFP OE-*E1*, *Sall2 KO1*, and WT ESCs with or without DOX induction. Relative to *Gapdh* expression (*n* = 3, technical replicates). Statistical significance was determined by two-way ANOVA with Tukey’s test, indicating significant changes between REX1:GFP OE-*E1* cells with (*E1*+) and without (*E1*−) DOX induction. ****P < 0.0001. **I** Flow cytometry analysis of SOX1:GFP during monolayer neural differentiation of SOX1:GFP OE-*Tuba1a*, *Sall2 KO3*, and WT ESCs with or without DOX induction. Relative to *Gapdh* expression (*n* = 3, technical replicates). Statistical significance was determined by two-way ANOVA with Tukey’s test, indicating significant changes between SOX1:GFP OE-*Tuba1a* cells with (*Tuba1a*+) and without (*Tuba1a*−) DOX induction. ****P < 0.0001. **J** Expression of neural markers (*Nestin*, *Pax6*, *Tubb3*) during monolayer neural differentiation of SOX1:GFP OE-*Tuba1a*, *Sall2 KO3*, and WT ESCs with or without DOX induction. Relative to *Gapdh* expression (*n* = 3, technical replicates). Statistical significance was determined by two-way ANOVA with Tukey’s test, indicating significant changes between SOX1:GFP OE-*Tuba1a* cells with (*Tuba1a*+) and without (*Tuba1a*−) DOX induction. ****P < 0.0001. **K** Immunofluorescence of TUBB3 at day 8 during monolayer neural differentiation of SOX1:GFP OE-*Tuba1a*, *Sall2 KO3*, and WT ESCs with or without DOX induction. The cells were immunostained for TUBB3. DAPI stained nuclei. Scale bar, 250 μm. **L** Phase images of NSCs derived from SOX1:GFP OE-*Tuba1a*, *Sall2 KO3*, and WT ESCs with or without DOX induction. Scale bar, 250 μm. **M** Flow cytometry analysis of SOX1:GFP^+^ cells during NTOs formation from SOX1:GFP OE-*Tuba1a*, *Sall2 KO3*, and WT ESCs with or without DOX induction at days 4, 5, 6 (*n* = 3, biological replicates). Statistical significance was determined by two-way ANOVA with Tukey’s test, indicating significant changes between SOX1:GFP OE-*Tuba1a* cells with (*Tuba1a*+) and without (*Tuba1a*−) DOX induction. ****P < 0.0001. **N** Flow cytometry analysis of SOX1:GFP/SOX2, SOX1:GFP/PAX6 positive cells at day 6 of NTOs formation from SOX1:GFP OE-*Tuba1a*, *Sall2 KO3*, and WT ESCs with or without DOX induction (*n* = 3, biological replicates). Statistical significance was determined by one-way ANOVA with Tukey’s test, indicating significant changes between SOX1:GFP OE-*Tuba1a* cells with (*Tuba1a*+) and without (*Tuba1a*-) DOX induction. ****P < 0.0001.

SALL2 was well known as a transcription factor to modulate gene transcription [51]. To figure out the potential downstream targets of SALL2 in regulating early neural differentiation, we constructed a hemagglutinin (HA)-tagged *Sall2* (*HA*-*Sall2*) ESC line to perform ChIP-seq. ChIP-seq profiles obtained with HA-tag antibody were distinct from input samples around the transcription start site (TSS) (Fig. 6A), validating the efficiency of ChIP. In total, we identified 131 SALL2 targets, with their genomic distribution mainly located at promoters (41.98%), intergenic (12.21%), exon (9.16%), transcription termination site (TTS, 9.16%), and small nuclear RNA (snRNA, 7.63%) (Fig. 6B). Gene Ontology (GO) analysis revealed that SALL2 targets were enriched in chromatin or gene regulatory functions (nuclear chromatin, protein−DNA complex, nucleosome, DNA packaging complex, and RNA polymerase complex), regulation of translation (negative regulation of translation, cytosolic ribosome, cytosolic large ribosomal subunit). Other than the cellular machinery functions, GO terms related to nervous systems, such as myelin sheath and presynaptic cytosol, were also enriched (Fig. 6C). Next, we overlapped the SALL2 targets with genes related to neural differentiation, and found 14 potential candidate genes (*Apoe*, *Ddc*, *Fus*, *Gas5*, *Lef1*, *Malat1*, *Marcksl1*, *Myc*, *Pim1*, *Polr2a*, *Snhg1*, *Snord118*, *Terc*, *Tuba1a*) (Fig. 6D). Further literature review showed that tubulin alpha 1a (*Tuba1a*) was specifically expressed in the central nervous system (CNS) and peripheral nervous system (PNS) of mouse embryo at E13.5. Knock-down of *Tuba1a* reduced the production of neural progenitors, while overexpression of WT TUBA1A could promote neurogenesis [52, 53]. PIM1 was a cell cycle regulator that could be recruited as a lineage determinant by enhancer. Also, as one of the downstream targets of STAT3, PIM1 supported self-renewal and inhibited endoderm differentiation of ESCs [54, 55]. These studies suggested that *Tuba1a* and *Pim1* might play important roles in neurogenesis.

In order to validate whether SALL2 regulated *Tuba1a* and *Pim1* during neural differentiation, we first analyzed our ChIP-seq data and found that SALL2 bound to the promoter regions of *Tuba1a* and *Pim1* (Fig. 6E), which was confirmed by ChIP-qPCR (Fig. 6F). We next investigated mRNA expression of *Tuba1a* and *Pim1* during monolayer neural differentiation by qRT-PCR. *Sall2* KO inhibited the expression of *Tuba1a* compared with the WT cells, but it barely affected *Pim1* expression (Fig. 6G and Supplementary Fig. S6A). After *E1 Sall2* induction, the expression of *Tuba1a* but not *Pim1* could be restored (Fig. 6H and Supplementary Fig. S6B). Therefore, we selected *Tuba1a* for further validation.

Next, we constructed DOX inducible *Tuba1a* expressing cell lines in SOX1:GFP *Sall2* KO ESCs (Supplementary Fig. S6C, D). We then examined *Tuba1a* expression during monolayer neural differentiation, which showed a similar level of *Tuba1a* to WT cells after DOX induction in *Sall2* KO cells (Supplementary Fig. S6E). Further flow cytometry analysis at different time points revealed that SOX1:GFP expression was also restored in the presence of *Tuba1a* (Fig. 6I). In addition, by qRT-PCR analysis, we found that the expression of *Nestin, Pax6*, and *Tubb3* inhibited by *Sall2* KO, was rescued by *Tuba1a* overexpression (Fig. 6J). We then collected differentiation day 8 cells to perform immunofluorescence staining of TUBB3, which displayed a comparable level with that of WT cells when *Tuba1a* was overexpressed (Fig. 6K).

Since *Sall2* KO ESCs failed to produce NSCs and decreased the NTOs forming efficiency, we determined the NSCs derivation and NTOs generation in *Tuba1a* overexpressing *Sall2* KO ESCs. After DOX induction, SOX2^+^ cells were detected in P1 NSCs, which could be further passaged (Fig. 6L and Supplementary Fig. S6F). For NTOs formation, in the presence of TUBA1A, the efficiency was significantly improved in SOX1:GFP *Sall2* KO cells, evidenced by morphology and flow cytometry analysis of SOX1:GFP (Fig. 6M and Supplementary Fig. S6G, H). In addition, the SOX1:GFP/SOX2 and SOX1:GFP/PAX6 positive cells were recovered to the levels of WT NTOs (Fig. 6N). Taken together, *Tuba1a* mediated SALL2 in regulating NSCs derivation and NTOs formation from ESCs.

## Discussion

As a member of the *Sal* family, *Sall2* was mainly documented as either a tumor suppressor or an activator [9]. Its role in development was less appreciated. In our study, by immunostaining, we mapped the SALL2 protein expression pattern from the zygote to the E15.5 mouse embryo, which was mostly consistent with *Sall2* mRNA expression. Though the self-renewal of ESCs was not affected by *Sall2* KO, the lineage differentiation, in particular, neural differentiation, was greatly impaired. Moreover, without *Sall2*, NSCs could not be derived from ESCs and the formation of NTOs was severely impacted as well. With ChIP-Seq, *Tuba1a* was verified as a SALL2 target in neural differentiation.

SALL2 protein was first detected in the epiblast of E5.5 embryo, indicating that *Sall2* was dispensable during pre- and peri-implantation stage embryo, which was also reflected by the fact that *Sall2* deficient ESCs maintain self-renewal and de nova ESC lines were established from *Sall2* KO blastocysts. As primed EpiSCs were derived from E5.5-E7.5 embryos and *Sall2* was considered as a marker gene of formative or primed PSCs [56], it will be worth interrogating its role in these stem cells.

In adult mouse brain neurons and human conjunctival epithelial cells, SALL2 has been detected in the cytoplasm by interacting with the p75 neurotrophin receptor (NTR), and upon nerve growth factor (NGF) treatment, SALL2 could be translocated into the nucleus [23, 51]. We noticed that as early as E11.5, while the nuclear-localized SALL2 mainly co-expressed with SOX2 in neural cells, in some TBBB3^+^ neurons, SALL2 appeared in the cytoplasm. Whether it resulted from reduced local NGF, increased p75NTR expression, or was related to the maturation of neurons warrants further investigation.

Our SALL2 immunostaining and previous ISH assay revealed its broad expression in the developing nervous system. By performing ESCs-based in vitro neural differentiation, we demonstrated the critical role of SALL2 in neural lineage commitment. *Sall2* deficiency in ESCs drastically reduced SOX1^+^ neural progenitors and subsequent TUBB3^+^ neurons, which, to some extent, mirrored the observed defects in NT and eye development in *Sall2* KO mice and patients with SALL2 mutations [11]. Notably, for the known two isoforms of *Sall2* [10], only E1 SALL2 restored neural differentiation in *Sall2* KO cells. The dominant expression of E1 SALL2 during neural differentiation may be linked to its function. Meanwhile, structurally, the nuclear localization sequence (NLS) and repression domain, which can interact with NuRD complex, in E1 SALL2 only may contribute to the functional difference between these two isoforms in neural differentiation [9]. It will be interesting to elucidate the function of the N-terminal domain of E1 SALL2 in the future.

NSCs are essential for the development and repair of the nervous system [57]. Strikingly, we could not derive NSCs from *Sall2* KO ESCs. Meanwhile, in the presence of *E1 Sall2* transgene, NSCs were obtained, but died after shutting off transgene expression. These findings demonstrated the critical role of SALL2 in the derivation and maintenance of NSCs from ESCs. In glioblastoma, SALL2, together with SOX2, POU3F2, and OLIGO2, reprogrammed differentiated tumor cells into tumor stem cells, and also, SALL2 was one of the partners of SOX2 [14], suggesting SALL2 may be essential for the homeostasis of NSCs in the nervous system in vivo as well.

The advance in NTO technology made it feasible for us to investigate SALL2’s function in NT development in vitro. By leveraging the NTO model, we successfully replicated the NTDs in *Sall2* deficient mice by showing a reduced number of NTOs, low-key marker gene expression and functional rescue by *E1 Sall2* expression. Since in mouse, compound KO of *Sall1*, *2*, and *4* resulted in severe NTDs [11], the compensatory and redundant function of these three *Sal* family members could be addressed with NTOs in future studies. Ocular coloboma is the defect manifested in patients with *SALL2* mutation and is also observed in *Sall2* deficient mouse embryo [13]. The retina organoids could be applied for the mechanistic study of *SALL2* in eye development, which may lead to potential therapeutic strategies for a clinic.

By overlapping SALL2 ChIP-seq targets with genes related to neural differentiation, we identified *Tuba1a* as a potential downstream target of SALL2. *Tubulin* genes are critical for cerebral cortex formation, and the mutations of various Tubulin isoforms have contributed to tubulin-related cortical dysgenesis or “Tubulinopathies” [58]. *Tuba1a* is one of the tubulin isoforms, which is highly expressed in neurons, but not in astrocytes or oligodendrocytes, during the development of nervous system and its expression was significantly downregulated after birth [59]. Over 70 *Tubua1a* mutations have been reported in a broad range of brain abnormalities such as lissencephaly and microencephaly etc. [60, 61]. Both the direct binding of SALL2 to *Tubua1a* promoter and restored neural differentiation, NSCs derivation and NTOs phenotypes in *Sall2* KO cells by *Tubua1a* transgene expression strongly validated *Tuba1a* as a direct functional target of SALL2 in the developing nervous system. TUBA1A may also be a potential therapeutic target for ocular coloboma patients with SALL2 mutations.

Until recently, a few studies have investigated the role of *SALL2* in cancer, which acted as either an activator or repressor depending on tumor type. In the nerve system, SALL2 has been identified as stemness factor contributed to the tumorigenesis of glioblastoma [14, 15]. The critical function of SALL2 in neural differentiation and one of its targets *Tuba1a* verified in this study may offer potential therapeutic strategy for the diagnosis and treatment of glioblastoma in future.

In summary, by outlying the SALL2 protein expression pattern during embryo development, we complemented the SALL2 expression landscape in early development. Using ESCs as a model system, we demonstrated that *Sall2* is critical for neural differentiation and NTOs formation, and indispensable for the derivation and maintenance of NSCs from ESCs. The newly identified target of SALL2, *Tuba1a* will not only shed light on the mechanism of *Sall2* in neural development but also facilitate the discovery of potential drug targets for relevant neural diseases.

## Materials and methods

### Animals

Specific-pathogen-free (SPF) mice were kept in the animal facility at Shanghai East Hospital affiliated to Tongji University.

### Embryo collection

Female ICR mice (5–6 weeks old), purchased from Shanghai Laboratory Animal Research Center, were super-ovulated by injection of pregnant mare serum gonadotropin (PMSG, 5 IU/mouse), followed by human chorionic gonadotropin (hCG, 5 IU/mouse) 48 h later, and then paired with adult males. Six pregnant mice were used to collect zygotes from oviducts at 20 h post-hCG injection. Post-implantation embryos from E5.5-E15.5 were collected; for each embryonic stage, at least three pregnant mice were used.

### Zygotic deletion of *Sall2* with CRISPR/Cas9

For gene editing and zygote injection, 100 ng/μL Cas9 protein (Novoprotein, E365, China) was mixed with 200 ng/μL sgRNA (synthetized by GenScript company, New Jersey, USA). The mixture was injected into the cytoplasm of the zygote with well-recognized pronuclei at a volume of 1–3 pL. Microinjection was performed in a droplet of M2 medium containing 5 μg/mL cytochalasin B (CB) using a Piezo-driven micromanipulator (Prime Tech, New York, USA). Then, the injected embryos were cultured in a KSOM medium with amino acids. The sgRNA sequence for *Sall2* deletion is 5′ CCACCGGUAAUGGUGAUAAUGUUUUAGAGCUAGAAAUAGCAAGUUAAAAUAAGGCUAGUCCGUUAUCAACUUGAAAAAGUGGCACCGAGUCGGUGC 3′. The sequence (500 bp) around the sgRNA targeted site was amplified by PCR, then sequenced and analyzed on the website (https://ice.synthego.com/#/), the sum of the percentage of different gene editing was counted as the embryo KO efficiency.

### In vitro embryo culture and ESC derivation

The zygotes (one-cell) were cultured in KSOM, 1-cell, 2-cell, 4-cell, 8-cell, morula and blastocyst stage embryos were collected and fixed in 2.5% paraformaldehyde (PFA) at different time points.

To derive ESCs from blastocysts, we followed the previously published protocol [62]. Briefly, the blastocysts were cultured in M15 ESC medium (KnockOut DMEM, supplemented with 10 ng/mL LIF (leukemia inhibitory factor), 15% fetal bovine serum (FBS), 1 × glutamine–penicillin–streptomycin (GPS, Gibco, New York, USA), 0.1 mM β-mercaptoethanol (β-ME, Sigma, Texas, USA) in a six-well plate with mitomycin C treated mouse embryonic fibroblasts as a feeder layer. After 2–4 days, the blastocysts attached, and the cells grew out of the zona pellucid. Around days 8–10, ESC colonies formed and were dissociated into single cells with accutase, then seeded into gelatin-coated six-well plate with M15.

### Preparation of mouse embryo frozen and paraffin sections

The procedure for frozen section preparation was performed on ice. Mouse E7.5-E8.5 embryos were dissected in PBS with 0.5% FBS, fixed in 4% PFA for 20–30 min, then washed three times with PBS, 15 min each. Then the embryos went through graded concentrations of PBS/Sucrose (10, 20, 30%). Finally, the embryos were embedded in O.C.T. compound in a plastic mold, frozen on dry ice, and transferred to −80 °C for storage.

Protocol for paraffin section preparation was used for E9.5-E15.5 embryos. The embryos were fixed in 4% PFA, overnight and washed three times with PBS, 5 min each, then dehydrated with gradient ethanol (50%, 75%, 90%, 95%, 100%), cleared with xylene two times, 20 min each. The embryos were transferred to glass bottles with xylene and paraffin at the ratio of 1:1 for 30 min, then replaced in paraffin three times, 1 h each. The tissues were embedded in paraffin and kept at 4 °C.

### Whole-mount immunostaining of mouse embryo

Early embryos were performed immunostaining according to the published protocol [63] with minor modifications. Blastocysts were washed in PBS (Hyclone, Utah, USA), fixed in 2.5% PFA for 15 min at room temperature (RT); permeabilized in PBS/0.025% Triton X-100 for 30 min. E5.5-E7.5 embryos were washed in PBS (Hyclone), fixed in 4% PFA for 30 min at RT; permeabilized in PBS/0.025% Triton X-100 for 3 h. Then embryos were blocked in PBS/0.1% bovine serum albumin (BSA)/0.01% Tween-20/2% FBS for 1 h or more, RT, and incubated with primary antibodies in blocking solution at 4 °C overnight. Embryos were then washed with blocking solution three times, 15 min each, incubated with Alexafluor-conjugated secondary antibodies (Invitrogen, California, USA) for 1 h, RT, washed with blocking solution three times, 15 min each. After going through 25, 50, 75, and 100% glycerol, the embryos were mounted in a small drop of Vectashield with DAPI and covered with a coverslip. The antibodies used were listed in Supplementary Table S1.

### Immunostaining of cultured cells

Cells were fixed in 4% PFA, blocked, and permeabilized with 1% BSA and 3% goat serum in PBS with 0.1% Triton X-100, then incubated with primary antibodies at 4 °C overnight. After washing four times, 5 min each, the cells were incubated with Alexafluor-conjugated secondary antibodies in the dark for 1 h, RT and washed four times, 5 min each, then incubated with DAPI for 15 min, RT. The cells were observed, and images were captured with a fluorescence microscope (Leica DMI4000, Germany). The antibodies used were listed in Supplementary Table S1.

### Vector construction

Single guide RNA (sgRNA) (5′ GCUUUCUGUACCUGGUCCTU 3′) targeting *Sall2* 3’UTR was cloned into pKLV-PB-U6-gRNA-PGK-Blast-T2A-TagBFP vector. For generating ESC lines with HA-FLAG tag knocked into *Sall2* loci, 800 bp DNA fragments upstream and downstream of the sgRNA site were amplified by PCR to generate 5′arm and 3′arm for homologous recombination. 5′arm, 3′arm and HA-FLAG tag linked with PGK-Puromycin was cloned into 19T vector (Takara, Japan) to generate targeting construct.

The cDNAs of two *Sall2* isoforms, *E1*, *E1A*, and *Tuba1a* were amplified by PCR, then cloned into PB-TRE transposon under the control of DOX inducible Tet response element (TRE) using homologous recombination to generate respective vectors. The primers used were listed in Supplementary Table S2.

### Cell culture

Naïve ESCs were cultured in 2i/LIF comprising the Mek inhibitor PD0325901 (1 μM), GSK3 inhibitor CHIR99021 (3 μM), and LIF (10 ng/mL) in N2B27 medium [25]. Cells were passaged every 2–3 days by accutase dissociation.

ESCs were also cultured in M15 medium and passaged every 2–3 days with 0.25% trypsin and EDTA (TE) dissociation for maintenance. All cell lines used in this study were tested for mycoplasma contamination every month.

### Generation of *Sall2* knockout cell lines

When ESCs were grown to 60% confluence on gelatin-coated six-well plates in M15 medium. sgRNAs 2 μg each (sg1: 5′ CCGACCGAAUUCCUCGCUCACCA 3′; sg2: 5′ CCACCGGUAAUGGUGAUAAUUGG 3′) and 1 μg spCas9 plasmid were co-transfected into ESCs using Lipofectamine LTX (Invitrogen), by following the manufacturer’s protocol. Twelve hours after transfection, the medium was refreshed, and 10 μg/mL blasticidin was added. Three to four days after selection, all cells in the non-transfected control group died, and the transfected cells were dissociated into single cells with accutase. 3000 cells were seeded into a gelatin-coated 10-cm dish. After 7–10 days, the colonies were picked into a gelatin-coated 96-well plate for amplification and characterization.

### Generation of transgene overexpressing ESC lines

When *Sall2* KO cells (REX1:GFP ESCs, SOX1:GFP ESCs) were grown to 60% confluence on gelatin-coated six-well plates. PB-TRE constructs (*E1, E1A, Tuba1a*) 2 μg each, 1 μg PBEF1α-*Tet3G*, and 1 μg HyPBase were co-transfected into ESCs using Lipofectamine 3000 (Invitrogen). Twelve hours after transfection, the M15 medium was refreshed, and 1 μg/mL puromycin was added to start the drug screening. About 3–4 days later, all cells in the non-transfected control group died, and the transfected cells were dissociated with accutase and resuspended, about 3000 cells were plated into gelatin-coated 10-cm dish. When the clones grew up, they were picked into a gelatin-coated 96-well plate for expansion and characterization.

### Generation of HA-Tag-ESC lines

When E14Tg2a ESCs were grown to 60% confluence in gelatin-coated six-well plates. 19T-5arm-Flag-HA-PGK-Puro-3arm, sgRNA, and spCas9 were co-transfected into ESCs by Lipofectamine 3000, according to the manufacturer’s instructions. Twelve hours after transfection, the medium was refreshed with 1 μg/mL puromycin for 3 days, then the cells were dissociated and 3000 cells were plated into gelatin-coated 10-cm dish to further culture 7 days. ESC colonies were picked and expanded for characterization.

### Monolayer neural differentiation

The neural differentiation protocol was adapted from Ying et al., with some modifications [40]. ESCs cultured in M15 were dissociated with TE into single cells and counted. About 7 × 10^4^ cells were plated into gelatin-coated six-well plates in serum medium with 1 ng/mL LIF overnight (day 0), the next day, the medium was changed to N2B27 (day 1), then regularly refreshed every 2 days until day 8.

### EB differentiation

The EB differentiation was performed as previously reported [34, 36, 37, 64]. ESCs cultured in M15 were dissociated into single cells with TE and counted. A drop (20 μL) of M10 medium (KnockOut DMEM, supplemented with 10% FBS, 1 × GPS, 0.1 mM β-ME), containing 1000 ESCs, was plated on the lid of 10-cm dish, which was then turned over to create a hanging drop (day 0). Every two days, each hanging drop was supplemented with 5 μL fresh M10 until day 8.

In addition, RA was added into M10 medium to induce ectoderm differentiation. About 10 × 10^4^ ESCs were plated in non-adhesive 12-well plate with 1 mL M10 medium plus 0.1 μM RA. The plate was placed on a shaker in the incubator (100 rpm). Every 2 days, the medium was refreshed and samples were collected until day 8.

### NSCs derivation from ESCs

The protocol for the derivation of NSCs from ESCs was as described previously [65] with minor modifications. When ESCs were grown to 80% confluence, the cells were passaged into gelatin-coated six-well plates, and cultured with M15 minus LIF for 2–3 days. Then the cells were dissociated with accutase and 1 × 10^5^ cells were plated into low-adhesive 12-well plates with 1 mL NDEF medium (N2B27, 20 ng/mL FGF2, 20 ng/mL EGF) on a shaker at 1200 rpm for 3 days to form neurospheres (days 1–3). At day 4, the neurospheres were seeded into gelatin-coated six-well plate and cultured with NSC medium (DMEM/F12, N2, 10 ng/mL EGF, 10 ng/mL FGF2, 50 μg/mL BSA) for about 5 days. After the NSCs migrated out from the neurospheres, the spheres were removed, then the remaining cells were dissociated with accutase, and plated into gelatin-coated 12-well plate for expansion and characterization.

### NTOs formation

To generate NTOs, the protocol from Park et al., was applied [50]. ESCs cultured in 2i/LIF were dissociated into single cells with accutase, collected into a 1.5 mL tube, then centrifuged at 1200 rpm, 3 min. The cells were washed twice with PBS and resuspended with N2B27. About 1125 cells were embedded into a drop (10 μL) of matrigel in a non-coated 24-well plate, at 37 °C for 20 min, then supplemented with 500 μL NDEF for conversion of ESCs into EpiSCs for 2 days. On day 3, the medium was switched to N2B27 for neural differentiation. On day 6, the NTOs were collected for characterization. The long, narrow aggregate with a central cavity and expressing SOX1:GFP was defined as NTO, and the number of NTOs was counted accordingly.

### T7 endonuclease assay and genotyping

sgRNA and spCas9 co-transfected cells and non-transfected cells were dissociated into single cells with accutase, centrifuged at 1200 rpm for 15 min. Then, the medium was removed, and the lysis buffer was added to lyse the cells on ice. sgRNA-guided gene editing was verified by T7 Endonuclease assay (Beyotime, China), according to the manufacturer’s instructions. In brief, 500 bp fragments up- and down-stream of the sgRNA targeted site were amplified by genomic PCR. About 200 ng PCR product was mixed with T7 endonuclease reaction buffer, heated at 95 °C for 5 min, allowing the temperature down to 85 °C at 2 °C/s, then down to room temperature at 0.1 °C/s. The hybridized PCR products were incubated with 1 μL T7 endonuclease at 37 °C for 20 min. The digested product was separated by agarose gel electrophoresis with edited genes displaying bands with different sizes.

For DOX inducible transgene expressing ESC colonies, the genomic DNA was prepared and primers targeting transgenes (*E1*, *E1A*, *Tuba1a*) were designed to detect the integration of transgene (*E1*, *E1A*, *Tuba1a*) and Tet-on-3G. After genomic PCR, the products were analyzed by agarose gel electrophoresis. The expression of transgene (*E1*, *E1A*, *Tuba1a*) under 1 μg/mL DOX induction was confirmed by qRT-PCR and western blot. The primers used were listed in Supplementary Table S2.

### Flow cytometry analysis

To monitor the generation of NTOs, NTOs were washed with cold organoid rinsing buffer (DAXIANG, KC100141, China) for 20 min on ice, centrifuged at 2000 rpm for 15 min. After removal of the supernatant, the cells were dissociated into single cells with accuatse, centrifuged at 1500 rpm, 5 min, then fixed with 4% PFA for 15 min, RT. Cells were permeabilized with 0.1% PBSTr (Triton X-100) for 15 min, RT, blocked with 3% BSA in 0.1% PBSTr for 1 h, RT. Then the cells were incubated with anti-SOX2 antibody or anti-PAX6 antibody at 4 °C overnight. The next day, the cells were washed with 0.1% PBSTr three times, then incubated with fluorescence-conjugated secondary antibody for 1 h, RT. After washing with 0.1% PBSTr three times, the cells were harvested in PBS for analysis. Antibodies used were listed in Supplementary Table S1.

To monitor neural differentiation, at different time points, the cells were washed with PBS and dissociated into single cells with accutase, centrifuged at 1200 rpm, 3 min. The supernatant was discarded, and the cells were resuspended with PBS, then filtered through a 40-µm-cell strainer. BD FACS Arial II was used for analysis according to the manufacturer’s instructions. All flow cytometry data analysis was performed with Flowjo (version 10.4.0).

### Western blot

Cells were dissociated with accutase, collected, and lysed in RIPA buffer with protease inhibitor (Roche, Switzerland) on ice for 30 min. The cell lysate was centrifuged at 12,000 rpm, 30 min, 4 °C. The supernatant was transferred into a 1.5 mL tube and the protein concentration was measured by BCA method (Beyotime, China). About 30 μg protein of each sample was loaded onto NuPAGE 10% Bis-Tris gel (Thermo Scientific, Massachusetts, USA). After electrophoresis, the protein was transferred onto PVDF membrane (Millipore, Massachusetts, USA), blocked with 5% skimmed milk for 1 h, RT, washed with 1% TBSTr three times, 5 min each, then incubated with primary antibodies at 4 °C overnight. The next day, the blots were washed with 1% TBSTr three times, 5 min each, then incubated with fluorescence-conjugated secondary antibodies for 1 h, RT, and signals were detected with ChemiDoc Touch Imaging System (Bio-rad, California, USA). The images were analyzed by ImageJ (version 1.53a). The antibodies used were listed in Supplementary Table S1, and the western blot raw data were listed in the Original data file.

### Quantitative real-time PCR (qRT-PCR)

Cells were washed twice with PBS and lysed in Trizol reagent (Takara). After chloroform extraction, RNA was precipitated with isopropanol, washed with 75% ethanol, and dissolved in RNase-free water. The RNA concentration was measured with a Spectrophotometer (NanoDrop Technologies, Inc., DE, USA). The reverse transcription was carried out by following the instructions of HiScript III 1st Strand cDNA Synthesis Kit (Vazyme, China). qRT-PCR was performed using Taq Pro Universal SYBR qPCR Master Mix with specific primers on ABI QuantStudio™ 6 Flex (Thermofisher). *Gapdh* expression was used to normalize the gene expression. The gene expression between different groups was compared with the ΔΔCT method. The primers used are listed in Supplementary Table S2.

### Chromatin immunoprecipitation sequencing

ChIP was performed with the SimpleChIP® Plus Sonication Chromatin IP Kit (Cell Signaling Technology, Massachusetts, USA). When HA-FLAG tagged ESCs were grown up to 80% confluence, the cells were dissociated into single cells with accutase and counted. About 5.7 × 10^5^ cells were plated into a gelatin-coated 10-cm dish for monolayer neural differentiation. At differentiation day 2, 1% formaldehyde was added into the medium to cross-link chromatin for 10 min, RT and quenched with 125 mM glycine for 5 min, RT, with rotation. Then cytoplasmic and nuclear cleavage was performed on ice in order to expose chromatin. After cell pellets were lysed, sonication was performed at the condition of PIP 75, Duty Factor 10%, CPB 200 (Covaris M220, Massachusetts, USA), 1 × 10^6^ cells per 130 μL in the Covaris tube. After sonication, 5% of the input was collected for the later library construction. About 100 μL nuclease-free water, 6 μL of 5 M NaCl, and 2 μL 10 mg/mL RNAase A were added to 50 μL cell lysate, incubated at 37 °C for 30 min. After removing RNA, 2 μL proteinase K was added to the fragmented chromatin and incubated at 65 °C for 2 h. DNA was purified and eluted to examine sonication quality. The chromatin was precleared and then immunoprecipitated with Protein A + G Magnetic beads coupled with HA-tag antibody (Supplementary Table S1). DNA fragments were purified and the libraries were constructed with VAHTS Universal DNA Library Prep Kit for Illumina V3 (Vazyme). Sequencing was performed by Shanghai Genefund Biotech Co., Ltd.

### ChIP-seq analysis

Raw reads were trimmed by TrimGalore! (version 0.6.4_dev) with default parameters in the paired-end (PE) mode to remove adapters and low-quality bases. Clean reads were then mapped to the mouse reference genome GRCm38 using Bowtie2 (version 2.4.2) with the parameter “--very-sensitive”. Reads in the blacklist regions were removed by bedtools (version 2.29.2). PCR duplicates were removed by Sambamba (version 0.7.1) [66]. Samtools (version 1.7) was applied to remove reads mapped to mitochondrial DNA [67]. Peaks were called by MACS2 (version 2.2.7.1) with parameters for narrow peak calling [68]. Peaks were annotated by Homer (version 4.11). GO was performed using the clusterProfiler (version 3.16.0) R package.

### Chromatin immunoprecipitation quantitative PCR (ChIP-qPCR)

After ChIP-seq analysis, potential targets were identified by ChIP-qPCR to determine the binding region of SALL2. According to the visualization of ChIP-seq data, ChIP-qPCR primers were designed from the SALL2 binding region of targets, and primers targeting the intergenic nonbinding region were used as negative control. DNA fragments were quantified with specific primers by qPCR assay (Takara). The values from the immunoprecipitated samples were normalized to 5% input DNA. The antibody used for ChIP was HA-tag (Supplementary Table S1), and the ChIP-qPCR primers were listed in Supplementary Table S2.

### Visualization of ChIP-seq data

To visualize the ChIP-seq data, we used deepTools (version 3.4.3) to transform clean BAM files to bigwig files through RPKM normalization and then generate the heatmap and average plots [69]. ChIP-seq signals were visualized in track view using Integrative Genomics Viewer (IGV, version 2.8.13) [70].

### RNA-seq analysis

The raw reads of public RNA-seq datasets were first trimmed by TrimGalore! (version 0.6.4_dev) with default parameters to remove adapters and low-quality bases. Clean reads were mapped to the mouse reference genome GRCm38 using Hisat2 (version 2.2.1) [71]. The featureCounts (version 2.0.1) was applied to quantify the reads to the exon level (-t exon) [72]. Reads were normalized to Transcripts Per Kilobase of exon model per Million mapped reads (TPM) by a compiled R script (R version 4.0.2) and then visualized by the “ggplot2” (version 3.4.1) R package.

### Statistical analysis

All data represented three biological replicates, three technical replicates, or where sample size (*n* ≥ 3) were indicated in the figure legends. Statistical analysis was conducted with Prism Version 9.0.1(128) (GraphPad). The normal distribution of data were confirmed by the Shapiro–Wilk test. Unpaired two-tailed Student *t*-test, one-way ANOVA, and two-way ANOVA were used for the comparison between groups. Statistical significance was defined as *P* value <0.05. Results were considered significant at *P < 0.05, **P < 0.01, ***P < 0.001, ****P < 0.0001.

## Supplementary information

Supplementary materials

Supplementary table S1

Supplementary table S2

Original data file

## Data Availability

The raw ChIP-seq data presented in this study have been deposited in the Genome Sequence Archive (GSA) in the National Genomics Data Center under the accession number CRA018705. Any additional information required to reanalyze the data reported in this paper is available from the corresponding authors upon reasonable request.
