# Supplementary material for: SALL2 regulates neural differentiation of mouse embryonic stem cells through Tuba1a
Source: Cell Death Dis. 2024 Sep 30;15(9):710. doi: 10.1038/s41419-024-07088-5 (PMC11442768; doi:10.1038/s41419-024-07088-5)
Supplement: Supplementary file 1 — Supplementary materials [file 41419_2024_7088_MOESM1_ESM.docx]

**SALL2 Regulates Neural Differentiation of Mouse Embryonic Stem Cells through *Tuba1a***

Hui Xiong^1,2,3,4,5,7,16^†, Bowen Lin^1,2,3,4,6,7,16^†, Junyang Liu^1,2,3,4,5,7,16^†, Renhong Lu^1,2,3,4,6,7,16^†, Zheyi Lin^1,2,3,4,6,7^, Chengwen Hang^1,2,3,4,6,7^, Wenjun Liu^8,9,10^, Lei Zhang^11,12^, Jie Ding^1,2,3,4,6,7^, Huixin Guo^13^, Mingshuai Zhang^1,2,3,4,5,7^, Siyu Wang^14^, Zheng Gong^1,14^, Duanyang Xie^1,2,3,4,6,7^, Yi Liu^1,2,3,4,6,7^, Dan Shi^1,2,3,4,7^, Dandan Liang^1,2,3,4,6,7,15^, Zhen Liu^8,9,10^, Yi-Han Chen^1,2,3,4,6,7, 15^* & Jian Yang^1,2,3,4,5,7,15^*.

^1^State Key Laboratory of Cardiovascular Diseases, Shanghai East Hospital, School of Medicine, Tongji University, Shanghai, 200120, China. ^2^Shanghai Arrhythmia Research Center, Shanghai East Hospital, School of Medicine, Tongji University, Shanghai, 200120, China. ^3^Department of Cardiology, Shanghai East Hospital, School of Medicine, Tongji University, Shanghai, 200120, China. ^4^Shanghai Frontiers Center of Nanocatalytic Medicine, Shanghai, 200092, China. ^5^Department of Cell Biology, School of Medicine, Tongji University, Shanghai, 200092, China. ^6^Department of Pathology and Pathophysiology, School of Medicine, Tongji University, Shanghai, 200092, China. ^7^Clinical Center for Heart Research, Tongji University, Shanghai, 200092, China. ^8^Institute of Neuroscience, CAS Center for Excellence in Brain Science and Intelligence Technology, CAS Key Laboratory of Primate Neurobiology, State Key Laboratory of Neuroscience, Chinese Academy of Sciences, Shanghai, 200031, China. ^9^Shanghai Center for Brain Science and Brain-Inspired Intelligence Technology, Shanghai, China. ^10^University of Chinese Academy of Sciences, Beijing, 100049, China. ^11^Department of Anatomy, Histology and Embryology, School of Medicine, Tongji University, Shanghai, 200092, China. ^12^Clinical center for brain and spinal cord research, School of Medicine, Tongji University, Shanghai, 200092, China. ^13^Department of Cardiology, the Second Hospital of Shanxi Medical University, Taiyuan, 030001, China. ^14^Jinzhou Medical University, Jinzhou, Liaoning, 121000, China. ^15^Research Units of Origin and Regulation of Heart Rhythm, Chinese Academy of Medical Sciences, Shanghai, 200092, China. ^16^These authors contributed equally: Hui Xiong, Bowen Lin, Junyang Liu, Renhong Lu.

Correspondence:

Yi-Han Chen ([yihanchen@tongji.edu.cn](mailto:yihanchen@tongji.edu.cn), ORCID: 0000-0003-1000-1835) or Jian Yang [(jy279@tongji.edu.cn, ORCID: 0000-0002-1964-7061)](mailto:(jy279@tongji.edu.cn,%20ORCID:%200000-0002-1964-7061))

**
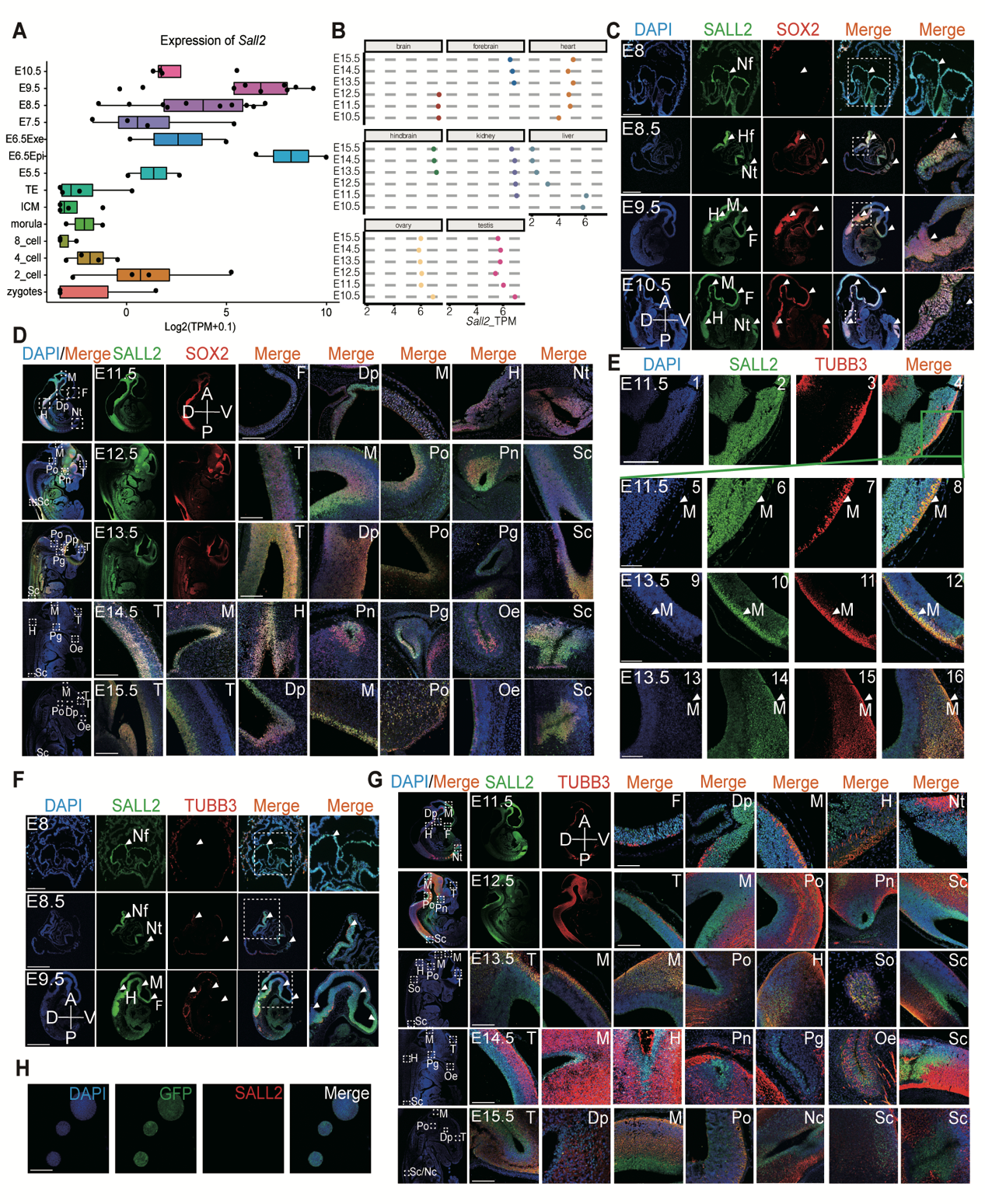
**

**Fig. S1:** **RNA-seq analysis of *Sall2* expression and immunofluorescence of SALL2, SOX2 and TUBB3 from zygote to E15.5 embryos.**

**A** RNA-seq analysis of *Sall2* expression in mouse E0.5-E10.5 embryos.

**B** RNA-seq analysis of *Sall2* expression in mouse E10.5-E15.5 embryos.

**C** Expression of SALL2 in mouse E8-E10.5 embryos. The embryos were immunostained for SALL2 and SOX2. DAPI stained the nuclei. The arrow heads indicated tissues with SALL2 expression. Dashed boxes indicated enlarged images. Scale bar, 250 μm.

**D** Expression of SALL2 in mouse E11.5-E15.5 embryos. The embryos were immunostained for SALL2 and SOX2. DAPI stained the nuclei. Dashed boxes indicated enlarged images. Scale bar, 250 μm.

**E** The cytoplasm localization of SALL2 in TUBB3^+^ neural cells. The embryos were immunostained for SALL2 and TUBB3. DAPI stained the nuclei. The arrow heads indicated tissues with SALL2 expression. Green square indicated enlarged images. Scale bar, 250 μm (1-4); 75 μm (5-16).

**F** Expression of SALL2 in mouse E8-E9.5 embryos. The embryos were immunostained for SALL2 and TUBB3. DAPI stained the nuclei. The arrow heads indicated tissues with SALL2 expression. Dashed boxes indicated enlarged images. Scale bar, 250 μm.

**G** Expression of SALL2 in mouse E11.5-E15.5 embryos. The embryos were stained for SALL2 and TUBB3. DAPI stained the nuclei. Dashed boxes indicated enlarged images. Scale bar, 250 μm.

**H** Expression of SALL2 in naïve ESCs by immunofluorescence. The cells were immunostained for SALL2. DAPI stained the nuclei. Scale bar, 250 μm. Acronym: Nf, Neural fold; Hf, Head fold; Nt, Neural tube; Forebrain; M, Midbrain; H, Hindbrain; Dp, diencephalon; Po, Pons; Pn, preoptic neuroepithelium; Sc, spinal cord; T, telencephalon; Oe, olfactory epithelium; So, Somite; Nc, notochord; A, Anterior; P, Posterior; D, Dorsal; V, Ventral.


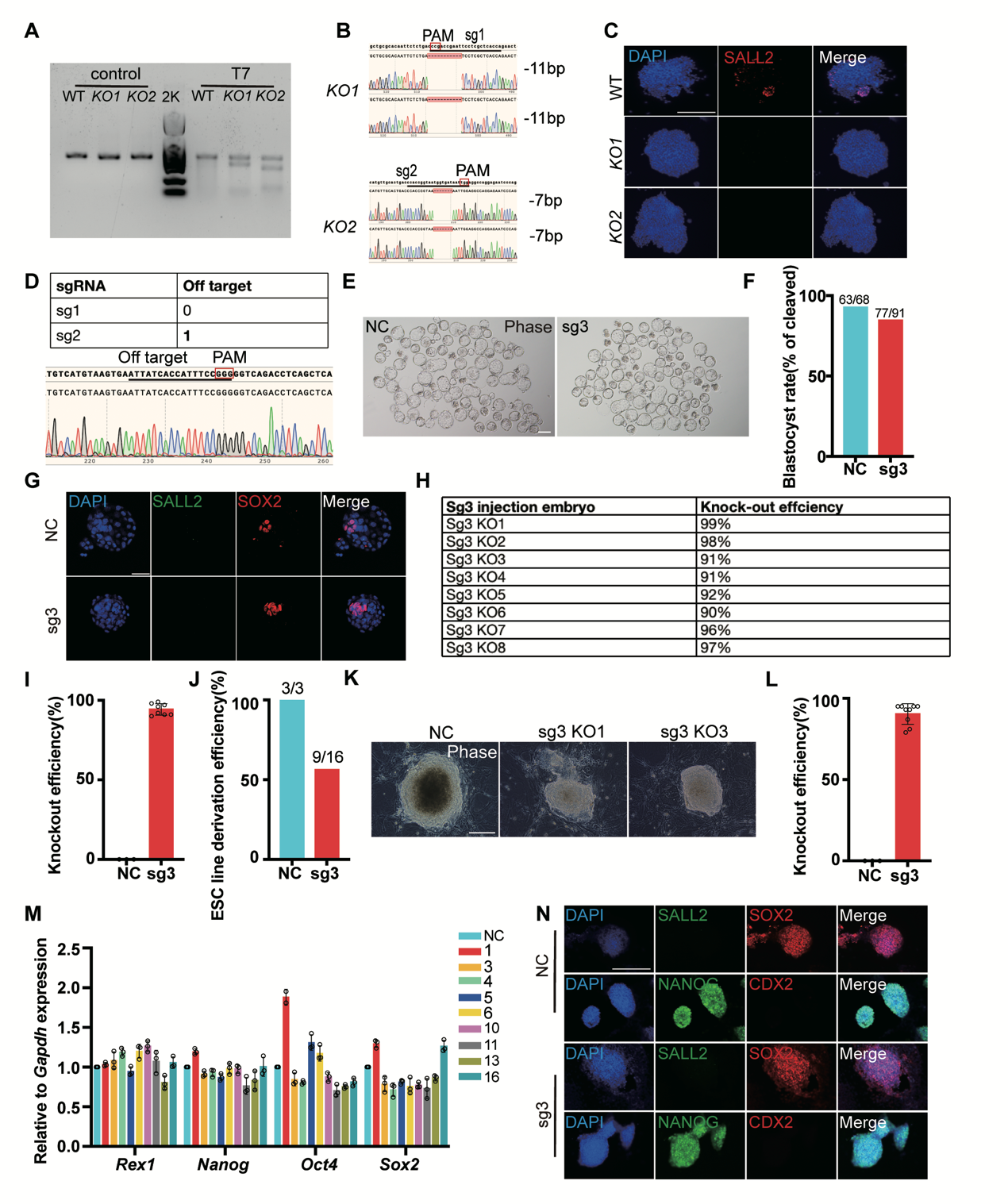


**Fig. S2:** ***Sall2* KO ESC lines were established from *Sall2* deficient blastocysts.**

**A** Agarose gel analysis of T7 endonuclease assay in REX1:GFP *Sall2* KO and WT ESCs.

**B** Sequencing of REX1:GFP *Sall2* KO ESCs. Red boxes indicated protospacer adjacent motif (PAM).

**C** Expression of SALL2 in REX1:GFP *Sall2* KO and WT ESCs. The cells were immunostained for SALL2. DAPI stained nuclei. Scale bar, 250 μm.

**D** Sequencing of predicted off targets of *Sall2* sgRNAs. Red box indicated PAM.

**E** Phase images of blastocysts developed from *Sall2* sgRNA injected (sg3) and noninjected (NC) zygotes. Scale bar, 100 μm.

**F** Efficiency of blastocyst formation from *Sall2* sgRNA injected (sg3) (n=91) and noninjected (NC) zygotes (n=68).

**G** Expression of SALL2 and SOX2 in blastocysts developed from *Sall2* sgRNA injected (sg3) and noninjected (NC) zygotes. The blastocysts were immunostained for SALL2 and SOX2. DAPI stained nuclei. Scale bar, 50 μm.

**H** KO efficiency of *Sall2* sgRNA injected (sg3) zygotes (n=8).

**I** Bar chart of KO efficiency of *Sall2* sgRNA injected (sg3) zygotes (n=8). Noninjected (NC) zygotes served as control (n=3).

**J** Efficiency of ESC line derivation from *Sall2* sgRNA injected (sg3) zygotes (n=9). Noninjected (NC) zygotes served as control (n=3).

**K** Phase images of ESC clones derived from *Sall2* sgRNA injected (sg3) and noninjected (NC) zygotes. Scale bar, 250 μm.

**L** Percentage of *Sall2* KO ESC clone derivation from *Sall2* sgRNA injected (sg3) zygotes (n=9). Noninjected (NC) zygotes served as control (n=3).

**M** qRT-PCR analysis of pluripotency markers (*Rex1*, *Nanog*, *Oct4*, *Sox2*) in *Sall2* KO ESC lines derived from *Sall2* sgRNA injected (sg3) zygotes. ESC line derived from noninjected (NC) zygote served as control. Relative to *Gapdh* expression (n=3, technical replicates).

**N** Expression of SALL2, SOX2, NANOG and CDX2 in *Sall2* KO ESC line derived from *Sall2* sgRNA injected (sg3) zygotes. ESC line derived from noninjected (NC) zygote served as control. The cells were immunostained for SALL2, SOX2, NANOG and CDX2. DAPI stained nuclei. Scale bar, 250 μm.


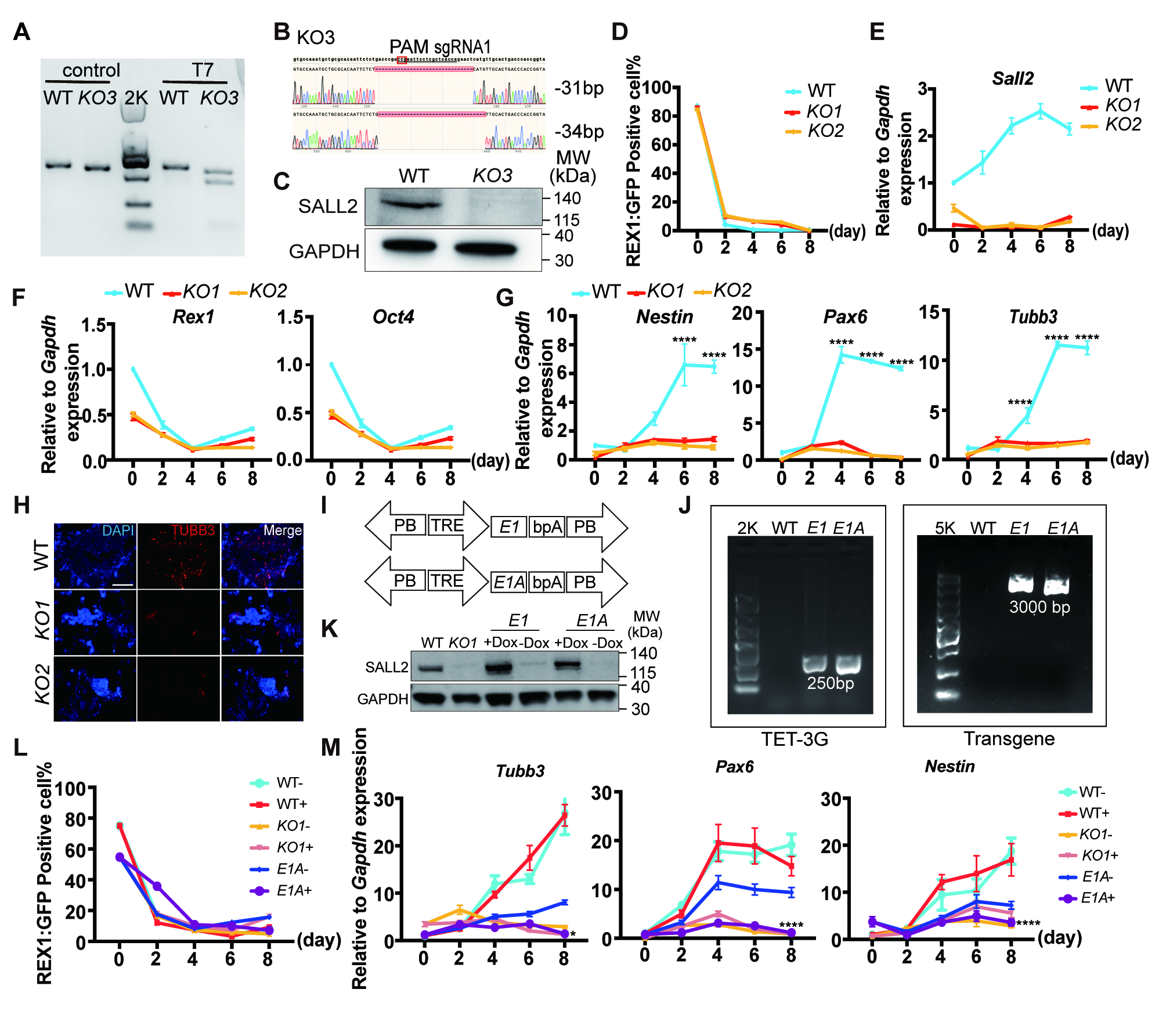


**Fig. S3:** **Construction and characterization of SOX1:GFP *Sall2* KO ESC lines and *Sall2* KO inhibited neural differentiation of ESCs.**

**A** Agarose gel analysis of T7 endonuclease assay in SOX1:GFP *Sall2* KO ESCs.

**B** Sequencing of SOX1:GFP *Sall2* KO ESCs. Red box indicated PAM.

**C** SALL2 Protein expression in SOX1:GFP *Sall2* KO and WT ESCs. GAPDH served as loading control.

**D** Flow cytometry analysis of REX1:GFP^+^ cells during monolayer neural differentiation of REX1:GFP *Sall2* KO and WT ESCs (n=3, biological replicates).

**E** *Sall2* expression during monolayer neural differentiation of REX1:GFP *Sall2* KO and WT ESCs. Relative to *Gapdh* expression (n=3, technical replicates).

**F** qRT-PCR analysis of pluripotency markers (*Rex1*, *Oct4*) during monolayer neural differentiation of REX1:GFP *Sall2* KO and WT ESCs. Relative to *Gapdh* expression (n=3, technical replicates).

**G** qRT-PCR analysis of neural markers (*Nestin*, *Pax6*, *Tubb3*) during monolayer neural differentiation of REX1:GFP *Sall2* KO and WT ESCs. Relative to *Gapdh* expression (n=3, technical replicates). Statistical significance was determined by two-way ANOVA with Tukey's test, indicating significant changes between *Sall2* KO cell lines (*KO1*, *KO2*) and WT cells. ****P < 0.0001.

**H** Expression of TUBB3 at day 8 during monolayer neural differentiation of REX1:GFP *Sall2* KO and WT ESCs. The cells were immunostained for TUBB3. DAPI stained nuclei. Scale bar, 250 μm.

**I** Schematic diagram of PB-TRE-*E1*, PB-TRE-*E1A* constructs.

**J** Agarose gel analysis of *E1*, *E1A* transgene and Tet-on-3G integration in REX1:GFP OE-*E1*, OE-*E1A* ESCs.

**K** Western blot analysis of SALL2 expression during monolayer neural differentiation of REX1:GFP OE-*E1*, OE-*E1A, KO1* and WT ESCs with or without DOX induction. GAPDH served as loading control.

**L** Flow cytometry analysis of REX1:GFP^+^ cells during monolayer neural differentiation of REX1:GFP OE-*E1A*, *KO1* and WT ESCs with or without DOX induction (n=3, biological replicates).

**M** qRT-PCR analysis of neural markers (*Tubb3*, *Pax6*, *Nestin*) during monolayer neural differentiation of REX1:GFP OE-*E1A*, *KO1* and WT ESCs with or without DOX induction at days 2, 4, 6, 8. Relative to *Gapdh* expression (n=3, technical replicates). Statistical significance was determined by two-way ANOVA with Tukey's test, indicating significant changes between REX1:GFP OE-*E1A* cells with (*E1A*+) and without (*E1A*-) DOX induction. *P < 0.05, ****P < 0.0001.


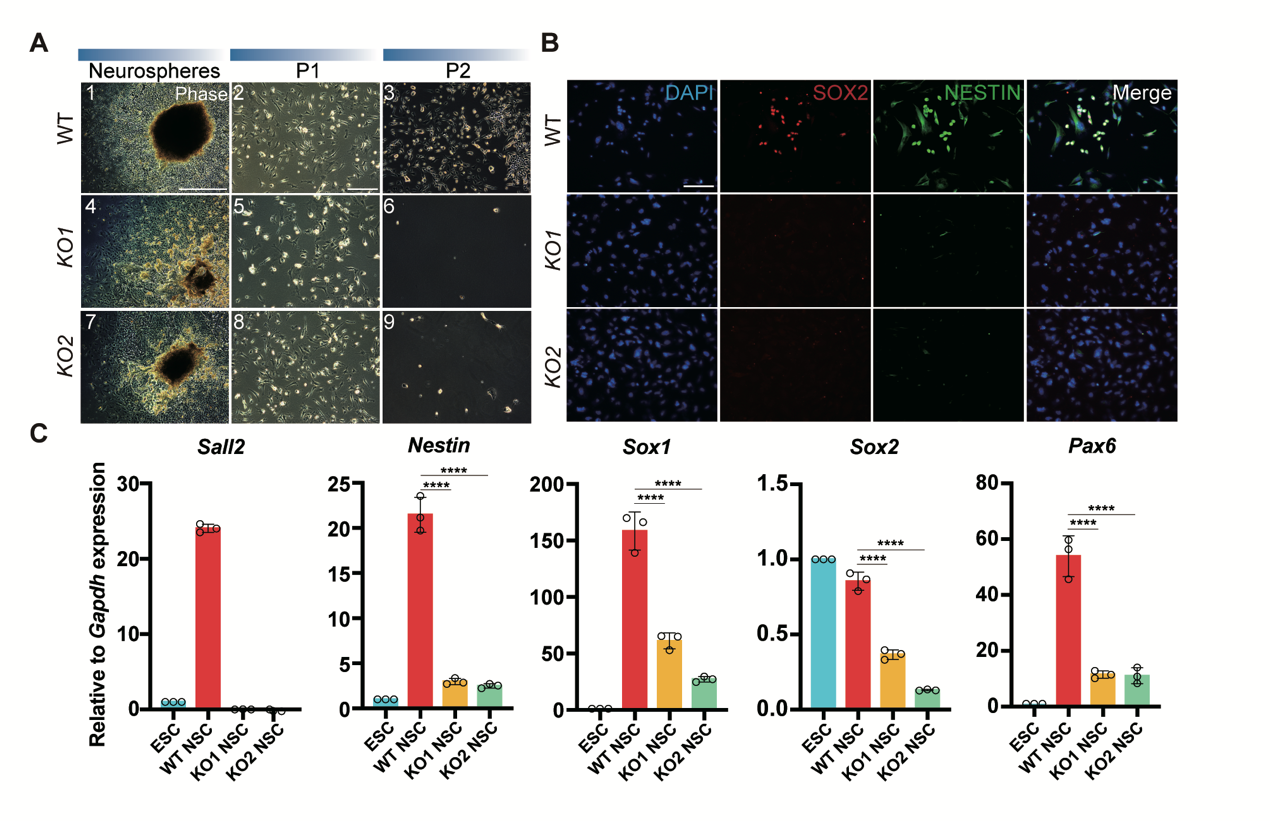


**Fig. S4:** ***Sall2* was indispensable for the derivation of NSCs from REX1:GFP ESCs.**

**A** Phase images of NSCs derivation from REX1:GFP *Sall2* KO and WT ESCs. Scale bar, 500 μm (1, 4, 7); 250 μm (2, 3, 5, 6, 8, 9).

**B** Expression of NESTIN and SOX2 in NSCs (P1) derived from REX1:GFP *Sall2* KO and WT ESCs. The cells were immunostained for NESTIN and SOX2. DAPI stained nuclei. Scale bar, 250 μm.

**C** qRT-PCR analysis of *Sall2* and NSC markers (*Nestin*, *Sox1*, *Sox2*, *Pax6*) in NSCs (P1) derived from REX1GFP *Sall2* KO and WT ESCs. Relative to *Gapdh* expression (n=3, technical replicates). Statistical significance was determined by one-way ANOVA with Tukey's test. ****P < 0.0001.


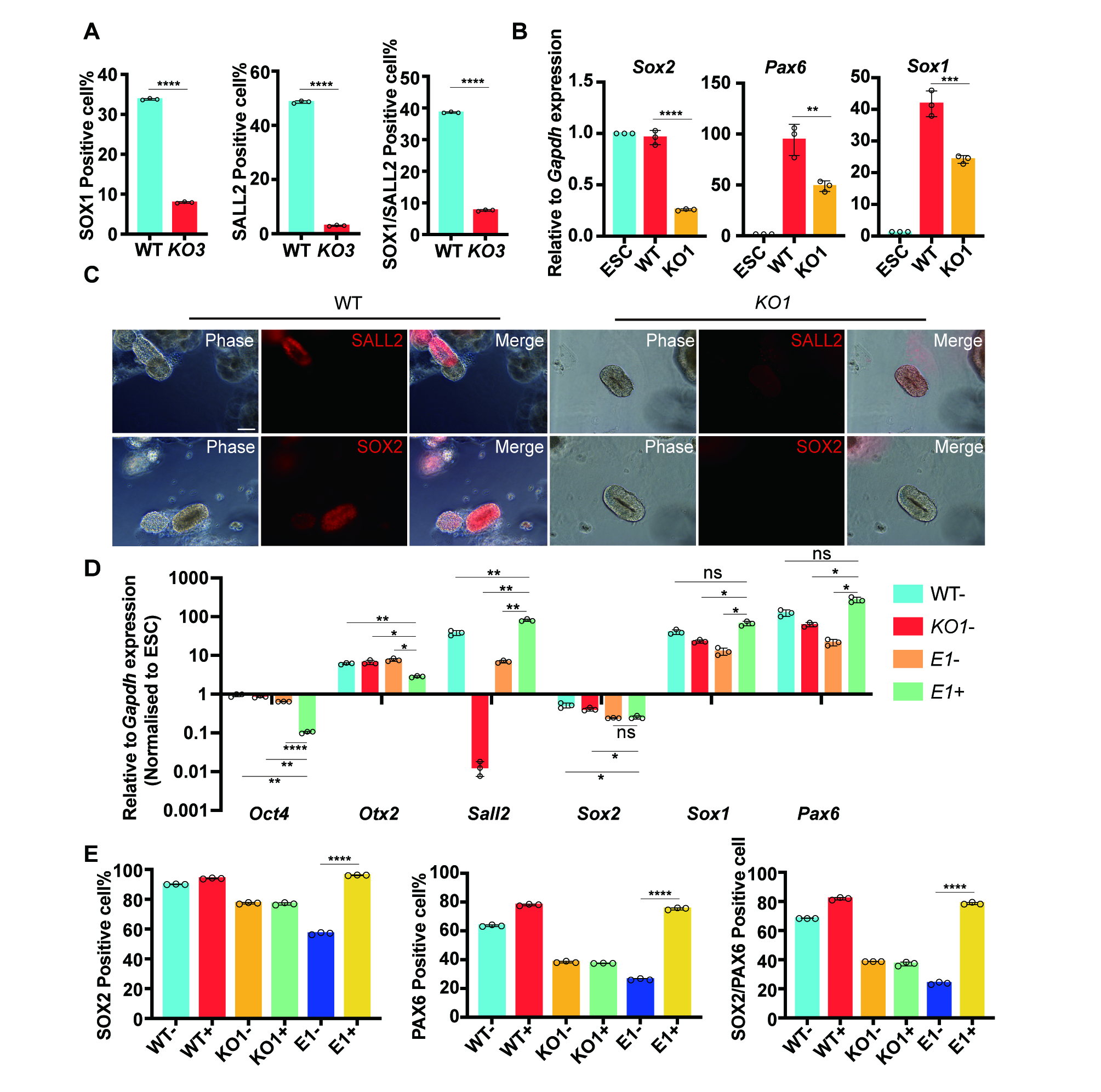


**Fig. S5:** ***E1* *Sall2* restored NTOs formation from *Sall2* KO ESCs**

**A** Flow cytometry analysis of SOX1:GFP^+^, SALL2^+^ cells during NTOs formation from SOX1:GFP *Sall2* KO and WT ESCs at differentiation day 6 (n=3, biological replicates). Statistical significance was determined by unpaired *t* test. ****P < 0.0001.

**B** qRT-PCR analysis of NTO markers (*Sox2*, *Pax6*, *Sox1*) during NTOs formation from REX1:GFP *Sall2* KO and WT ESCs at differentiation day 6. Relative to *Gapdh* expression (n=3, technical replicates). Statistical significance was determined by one-way ANOVA with Tukey's test. **P < 0.01, ***P < 0.001, ****P < 0.0001.

**C** Immunofluorescence of SALL2 and SOX2 expression in REX1:GFP *Sall2* KO and WT NTOs at differentiation day 6. The NTOs were immunostained for SALL2 and SOX2. DAPI stained nuclei. Scale bar, 250 μm.

**D** qRT-PCR analysis of *Sall2*, *Oct4*, *Otx2* and NTO markers (*Sox2*, *Sox1*, *Pax6*) during NTOs formation from REX1:GFP OE-*E1* (with and without DOX induction), REX1:GFP *Sall2* *KO1* and WT ESCs at differentiation day 6. Relative to *Gapdh* expression and normalized to ESCs (n=3, technical replicates). Statistical significance was determined by two-way ANOVA with Tukey's test. *P <0.05, **P <0.01, ****P <0.0001, ns, not significant.

**E** Flow cytometry analysis of SOX2^+^, PAX6^+^, SOX2/PAX6 positive cells during NTOs formation from REX1:GFP OE-*E1*, REX1:GFP *Sall2* *KO1* and WT ESCs with and without DOX induction at differentiation day 6 (n=3, biological replicates). Statistical significance was determined by one-way ANOVA with Tukey's test. ****P < 0.0001.


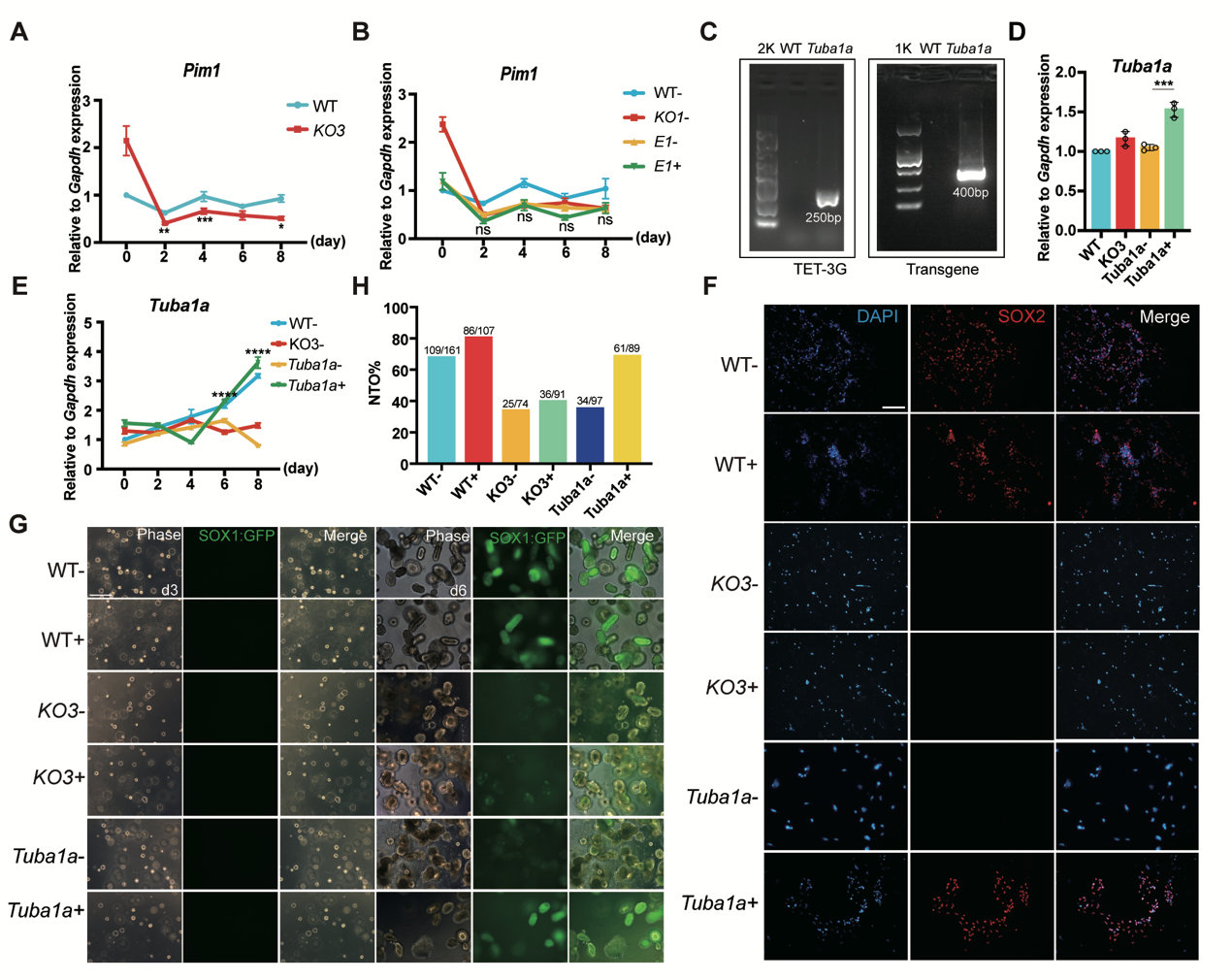


**Fig. S6: SALL2 regulated neural differentiation of ESCs through *Tuba1a*.**

**A** *Pim1* expression during monolayer neural differentiation of SOX1:GFP *Sall2* KO and WT ESCs. Relative to *Gapdh* expression (n=3, technical replicates). Statistical significance was determined by unpaired *t* test. *P <0.05, **P <0.01, ***P <0.001.

**B** *Pim1* expression during monolayer neural differentiation of REX1:GFP OE-*E1* (with and without DOX induction), *Sall2 KO1* and WT ESCs. Relative to *Gapdh* expression (n=3, technical replicates). Statistical significance was determined by two-way ANOVA with Tukey's test. ns, not significant.

**C** Agarose gel analysis of *Tuba1a* transgene and Tet-on-3G integration in SOX1:GFP OE-*Tuba1a* ESCs.

**D** mRNA expression of *Tuba1a* in SOX1:GFP OE-*Tuba1a* (with and without DOX induction), *Sall2 KO3* and WT ESCs. Relative to *Gapdh* expression (n=3, technical replicates). Statistical significance was determined by one-way ANOVA with Tukey's test. ***P <0.001.

**E** *Tuba1a* expression during monolayer neural differentiation of SOX1:GFP OE-*Tuba1a* (with and without DOX induction), *Sall2 KO3* and WT ESCs. Relative to *Gapdh* expression (n=3, technical replicates). Statistical significance was determined by two-way ANOVA with Tukey's test, indicating significant changes between SOX1:GFP OE-*Tuba1a* cells with (*Tuba1a*+) and without (*Tuba1a*-) DOX induction. ****P < 0.0001.

**F** Immunofluorescence of SOX2 expression in NSCs (Passage 1) derived from SOX1:GFP OE-*Tuba1a*, *Sall2* *KO3* and WT ESCs with or without DOX induction. The cells were immunostained for SOX2. DAPI stained nuclei. Scale bar, 250 μm.

**G** Phase and fluorescence images of NTOs generated from SOX1:GFP OE-*Tuba1a*, *Sall2* KO and WT ESCs at differentiation days 3 and 6. Scale bar, 250 μm.

**H** Number of NTOs generated from SOX1:GFP OE-*Tuba1a*, *Sall2* KO and WT ESCs.
