# Supplementary material for: SALL2 regulates neural differentiation of mouse embryonic stem cells through Tuba1a
Source: Cell Death Dis. 2024 Sep 30;15(9):710. doi: 10.1038/s41419-024-07088-5 (PMC11442768; doi:10.1038/s41419-024-07088-5)

Western Blot Relative to Fig. 2C

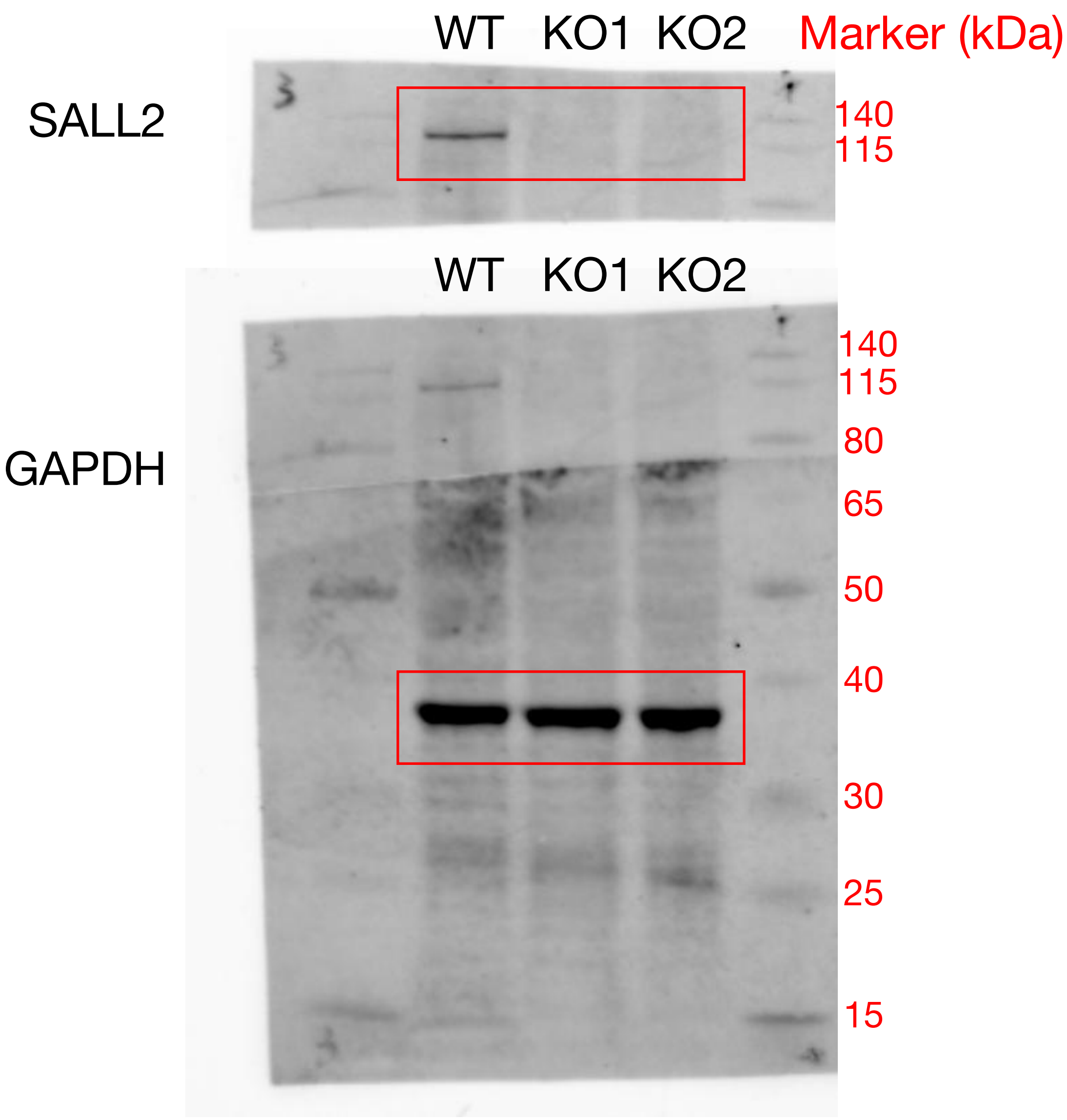

Western Blot Relative to Fig. 3I

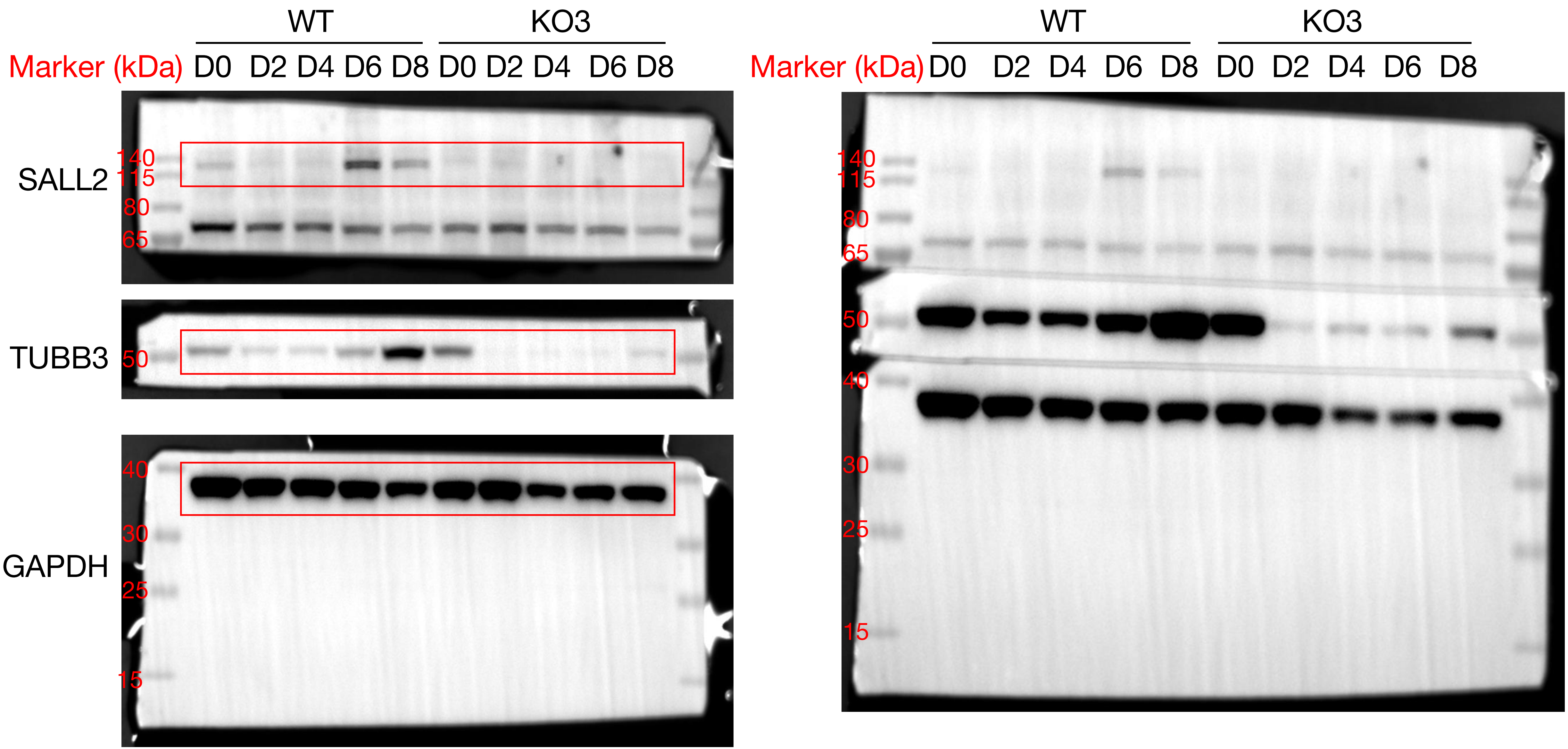

Western Blot Relative to Fig. 5F

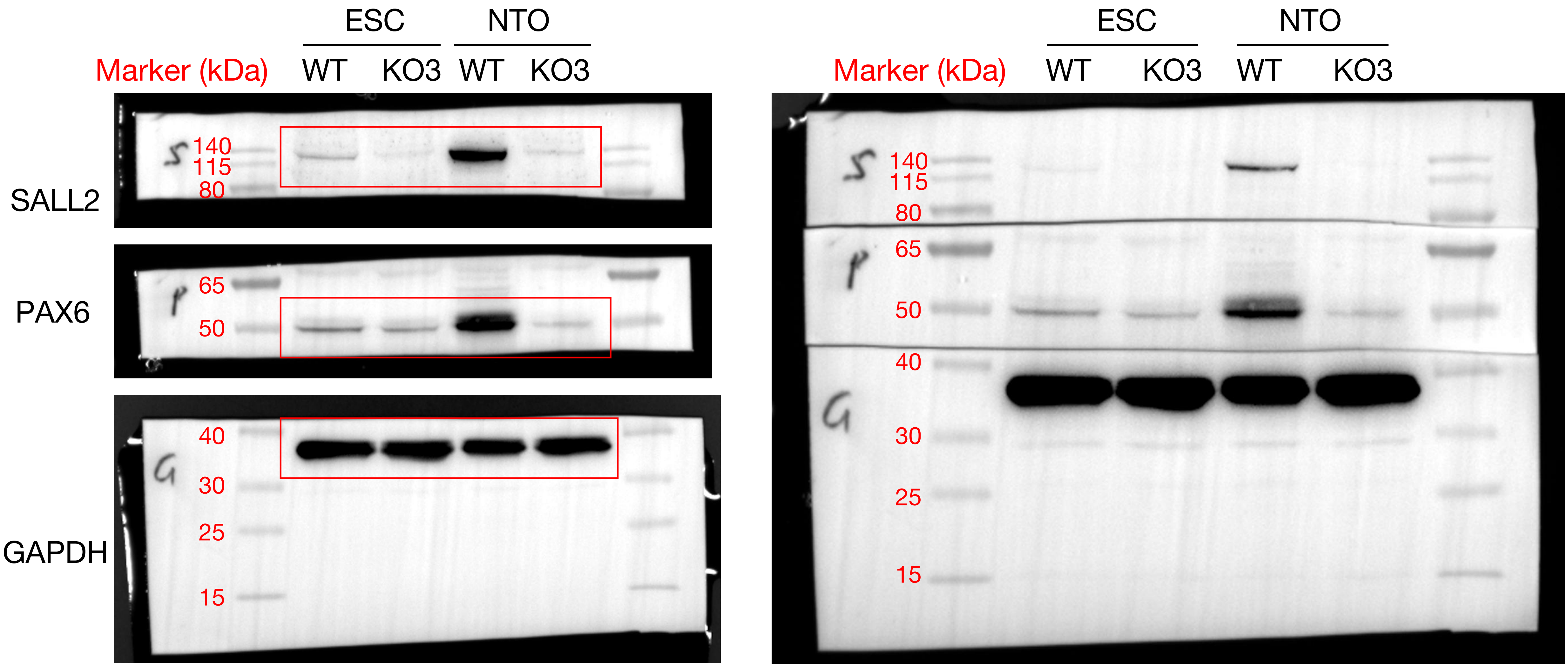

Western Blot Relative to Fig. S3C

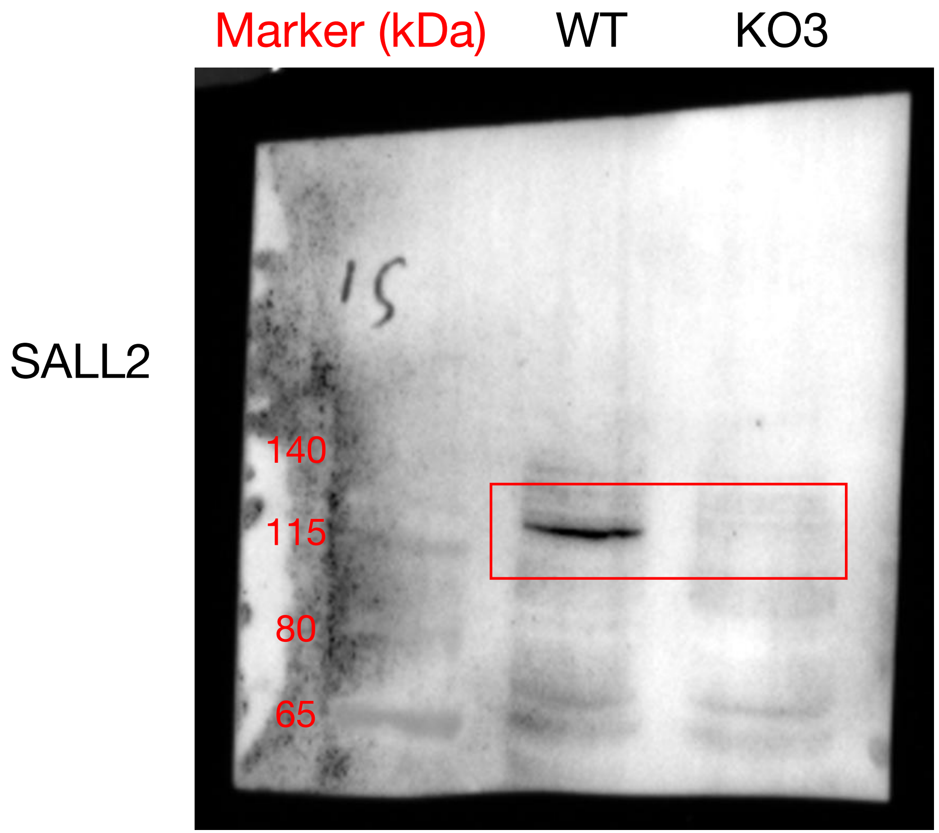

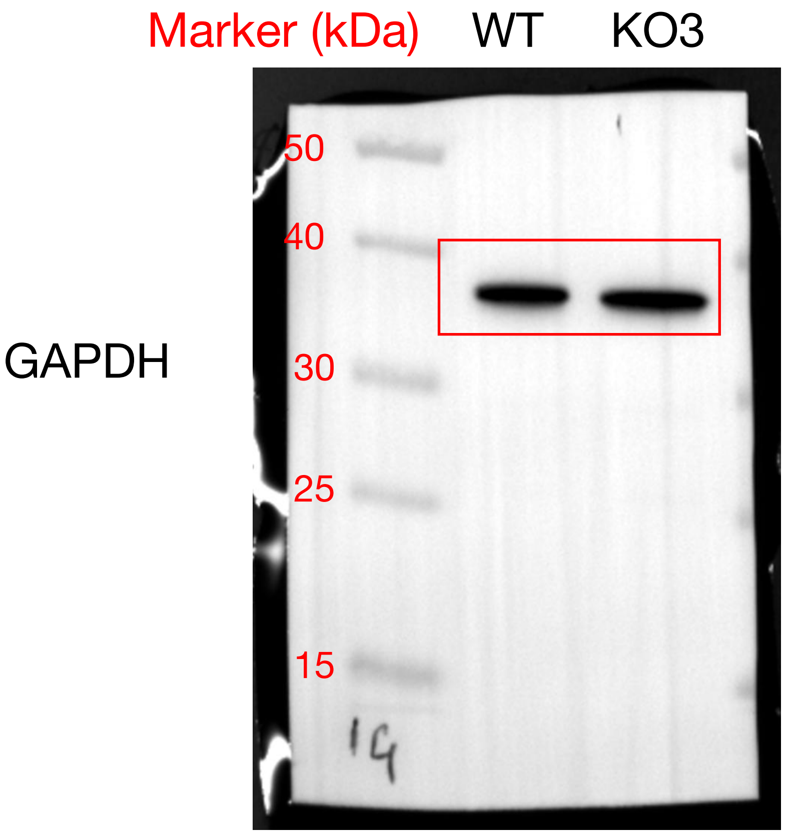

Western Blot Relative to Fig. S3K

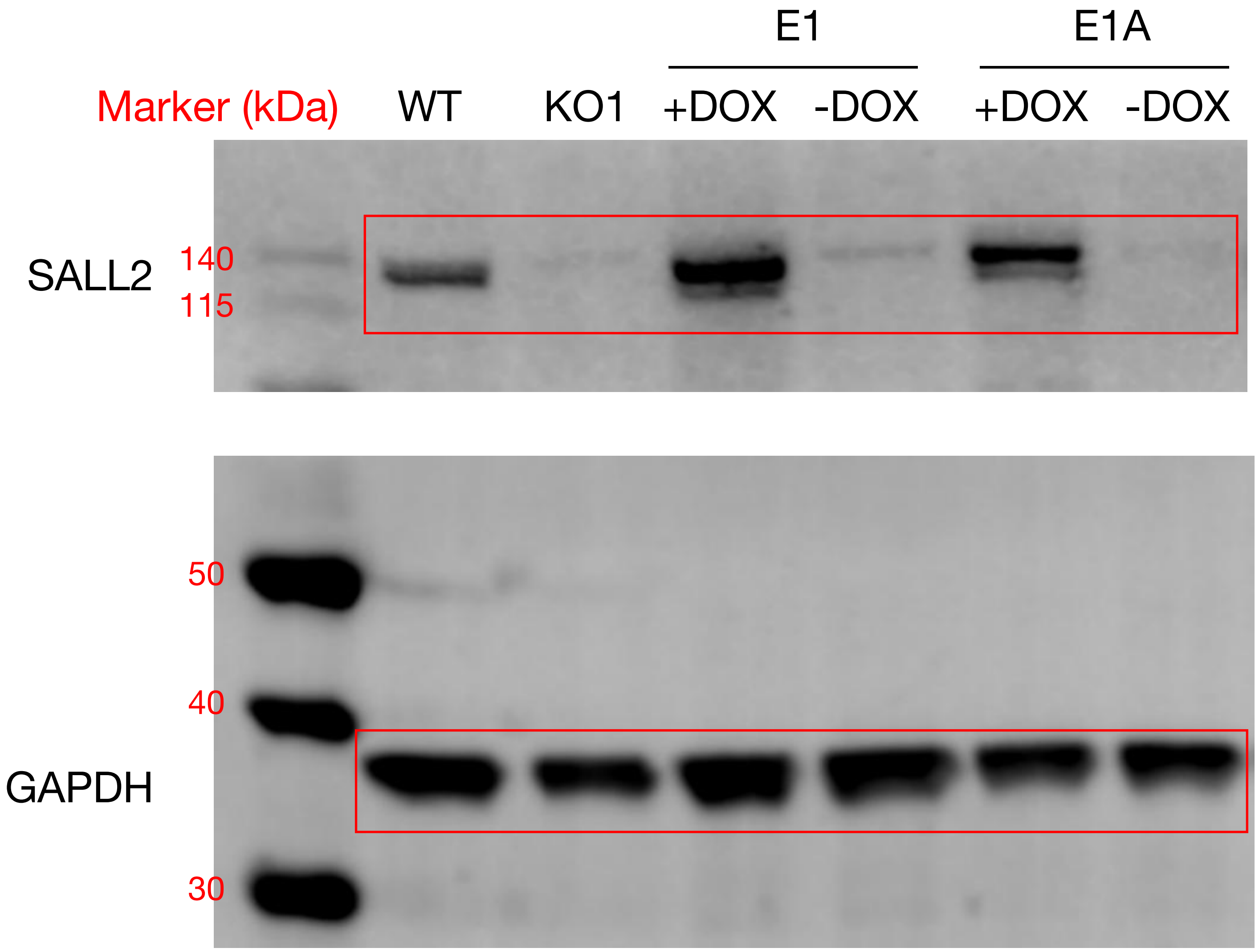

Supplement: Supplementary file 4 — Original data file [file 41419_2024_7088_MOESM4_ESM.pdf]
